# Supplementary material for: Evaluation of unsupervised learning algorithms for the classification of behavior from pose estimation data
Source: Patterns (N Y). 2025 Apr 22;6(5):101237. doi: 10.1016/j.patter.2025.101237 (PMC12142628; doi:10.1016/j.patter.2025.101237)
Supplement: Document S1. Figures S1–S8 and Tables S1 and S2 [file mmc1.pdf]

Patterns, Volume *6*

Supplemental information

Evaluation of unsupervised learning algorithms for the classification of behavior from pose estimation data

Jakub Mlost, Rame Dawli, Xuan Liu, Ana Rita Costa, and Iskra Pollak Dorocic

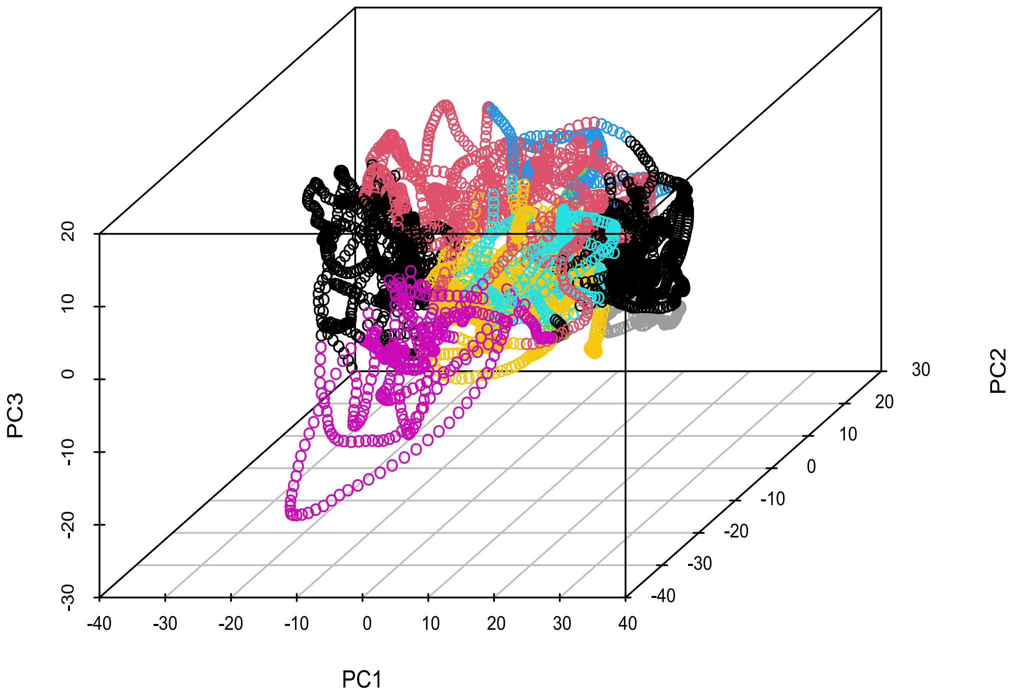

Figure S1. Visualization of the feature space from BFA package after PCA dimensionality reduction and K-means clustering. Related to second paragraph in **Clustering Algorithm** section and Figure 2.

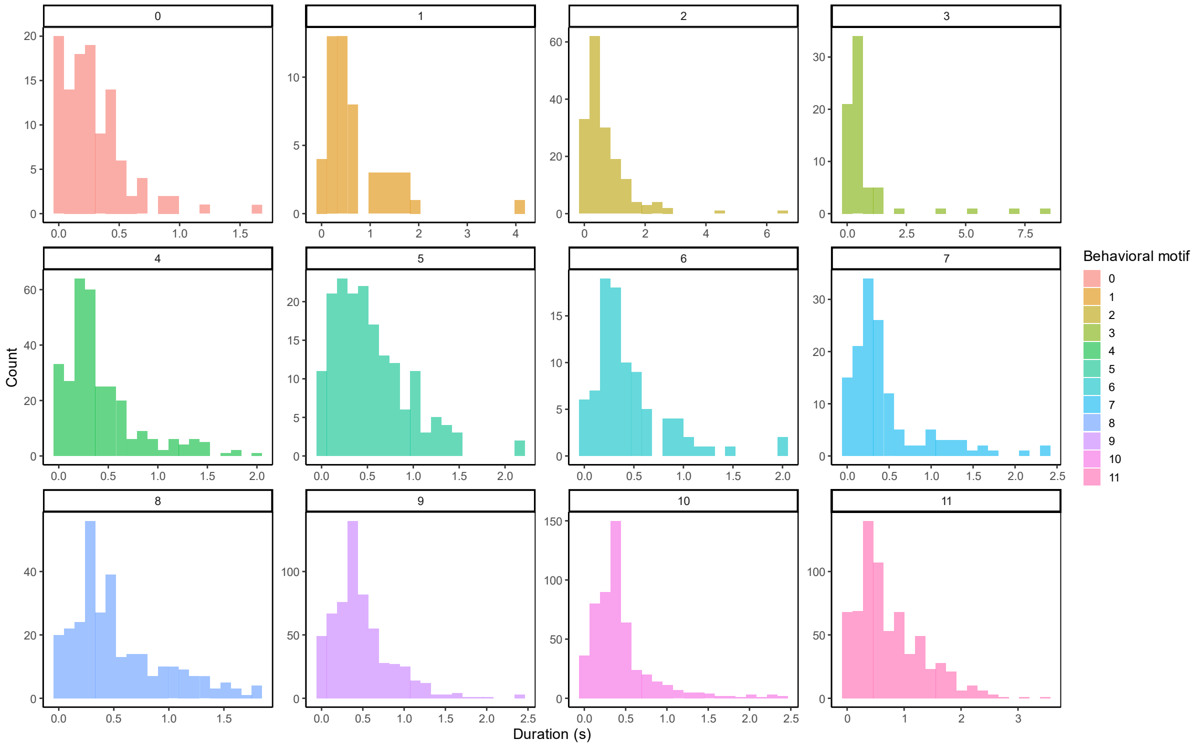

Figure S2. Frequency histograms of number of frames occurring for each behavioral motif produced by B-SOiD. The X-axis presents the duration of the occurrence, while Y axis represents the number of occurrences with a given duration. Values represent distributions across 3 samples of 10-minute recordings. Related to Figure 5.

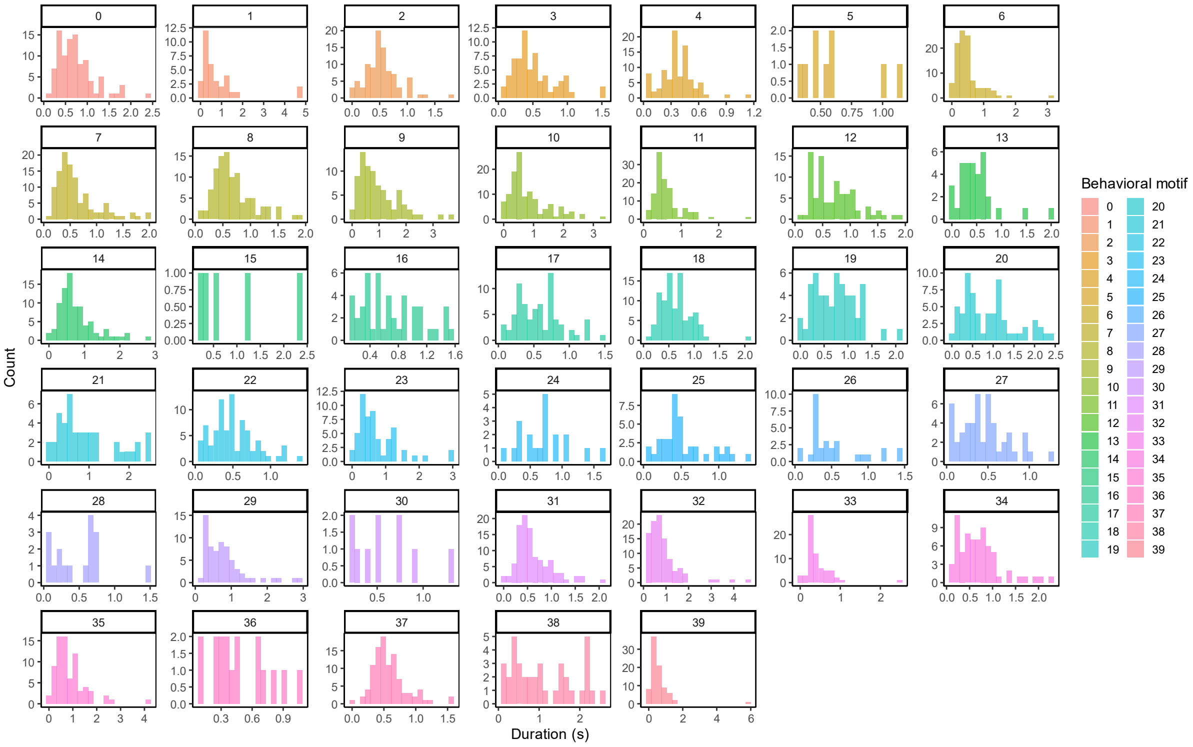

Figure S3. Frequency histograms of number of frames occurring for each behavioral motif produced by VAME. The X-axis presents the duration of the occurrence, while Y axis represents the number of occurrences with a given duration. Values represent distributions across 3 samples of 10-minute recordings. Related to Figure 5.

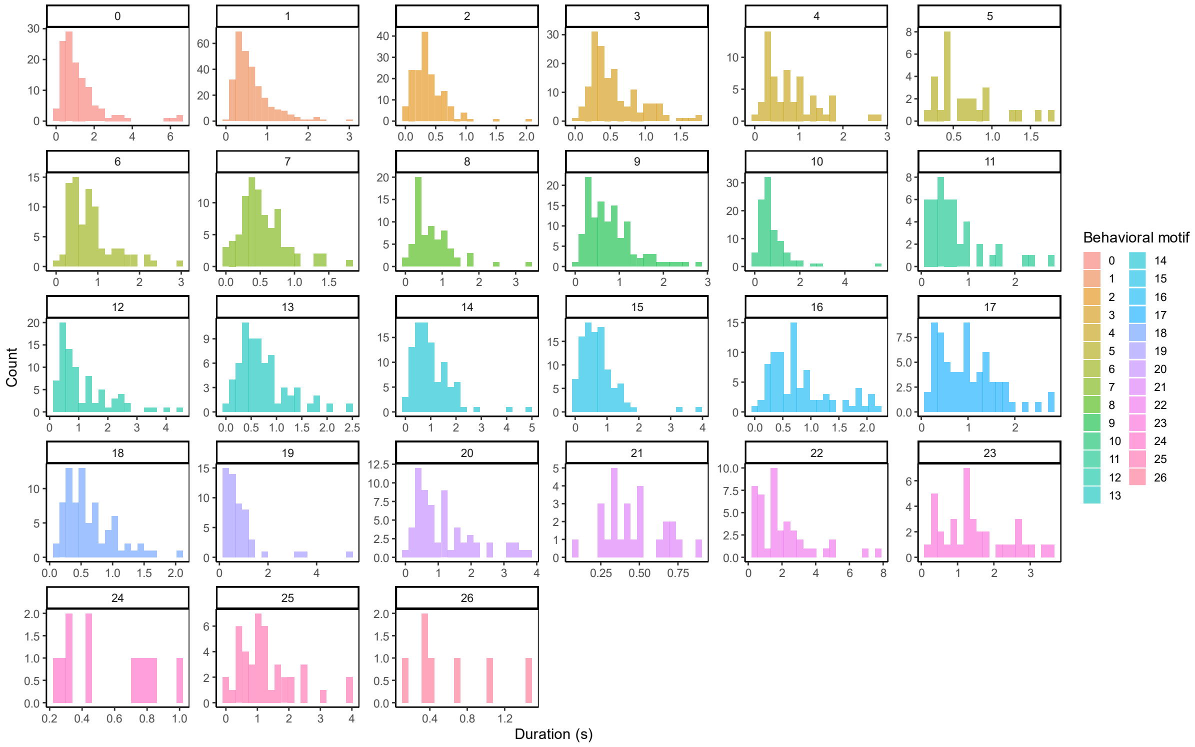

Figure S4. Frequency histograms of number of frames occurring for each behavioral motif produced by Keypoint-MoSeq. The X-axis presents the duration of the occurrence, while Y axis represents the number of occurrences with a given duration. Values represent distributions across 3 samples of 10-minute recordings. Related to Figure 5.

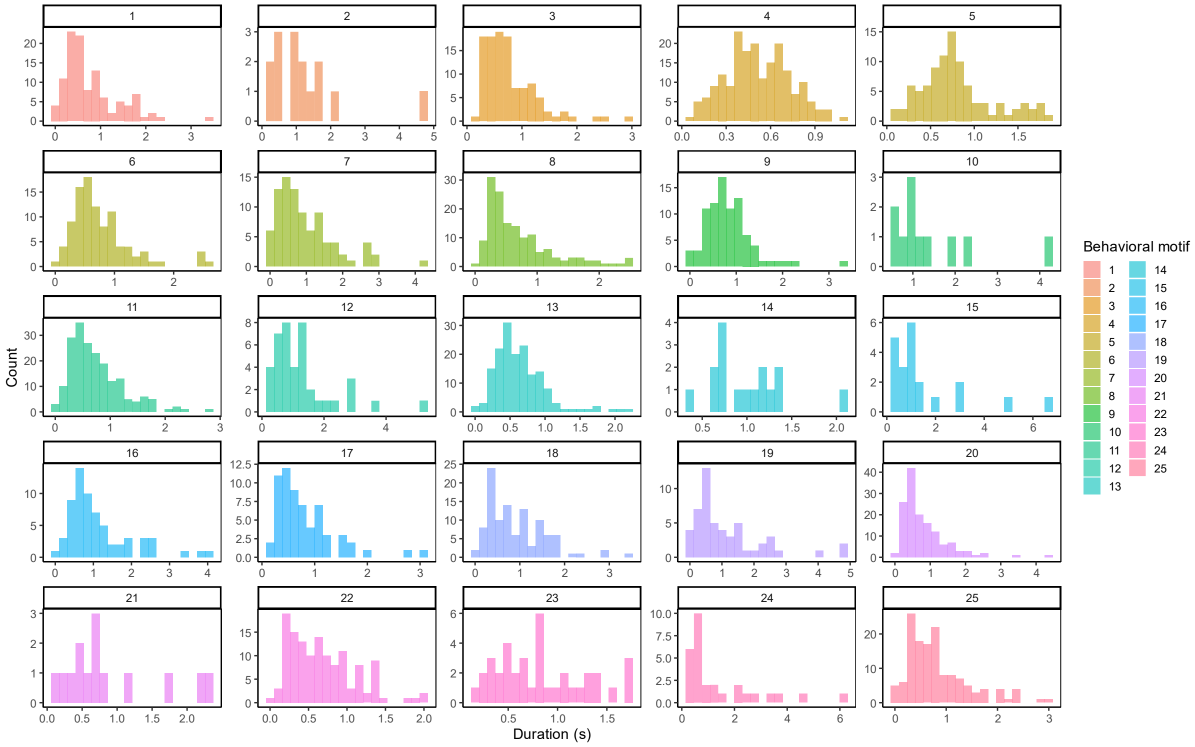

Figure S5. Frequency histograms of number of frames occurring for each behavioral motif produced by BFA. The X-axis presents the duration of the occurrence, while Y axis represents the number of occurrences with a given duration. Values represent distributions across 3 samples of 10-minute recordings. Related to Figure 5.

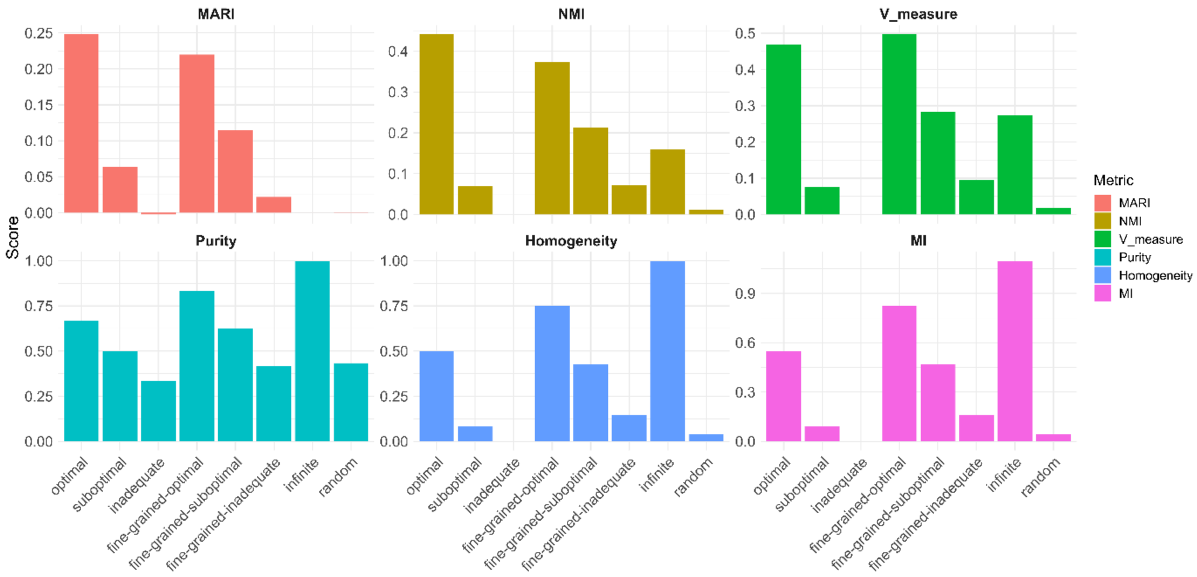

Figure S6. External validity measures scores for MARI (A), NMI (B), V-measure (C), Purity (D), Homogeneity (E) and Mutual Information (F) against mock behavioral data. Optimal solution represents presumed 100% accuracy in consistent clustering of behavioral motifs into more fine grained clusters. Suboptimal, inadequate and invalid solutions represents 75%, 50% and 25% in presumed accuracy against manual labeling data. Fine-grained counterparts are splitting mock manual labeling into a higher number of clusters. Inifite solution has as many clusters as data points. Related to Figure 6.

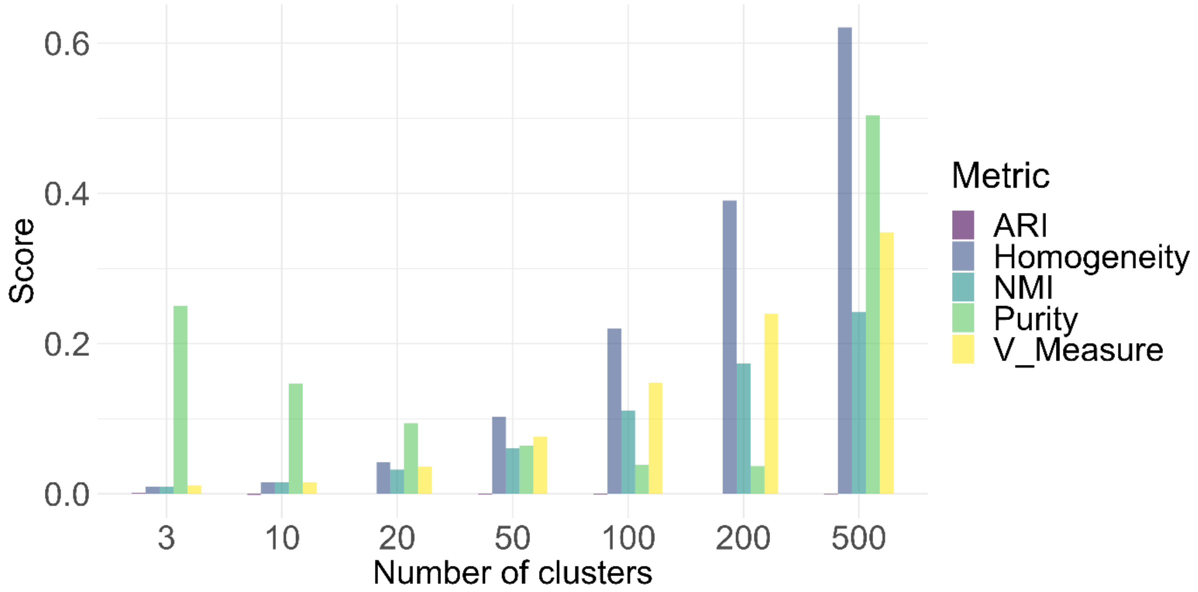

Figure S7. External validity measures scores for a random dataset of 1000 datapoints, split randomly into 10 groups and then assessed against random clustering, related to Figure 6**.**

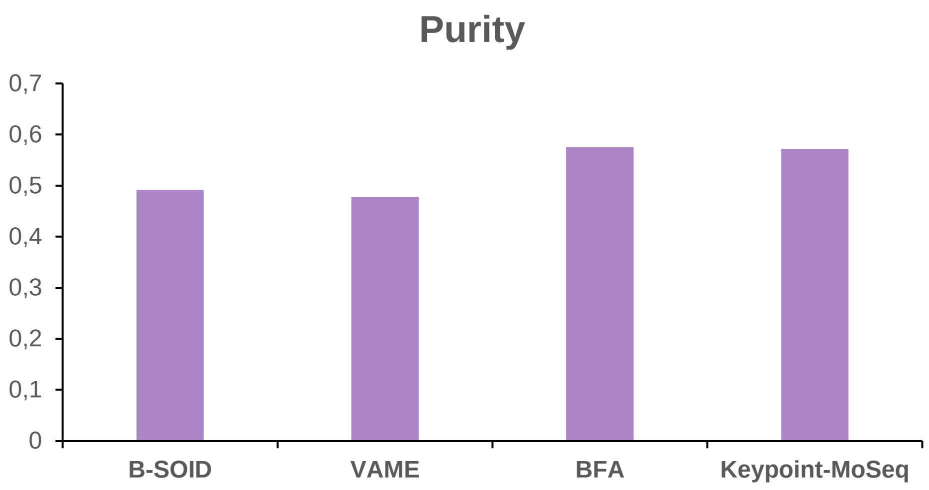

Figure S8. Purity measurement across different clustering algorithms. Positive values denote the best clustering efficacy, related to Figure 6**.**

Score	B-SOiD	VAME	BFA	Keypoint-MoSeq
Silhouette Score	0,240	-0,013	0,017	-0,267
MARI	0,196	0,077	0,157	0,202
Purity	0,491	0,477	0,575	0,571

Table S1. Numerical values for quantitative comparison of different methods, related to Figure 6.

Solution	Purity	MI	MARI	Homogeneity	NMI	V_measure
optimal	0,666	0,549	0,248	0,5	0,442	0,469
suboptimal	0,5	0,093	0,063	0,084	0,068	0,076
inadequate	0,333	0	0	0	0	0
fine-grained-optimal	0,833	0,823	0,22	0,75	0,372	0,498
fine-grained-suboptimal	0,625	0,466	0,115	0,425	0,212	0,283
fine-grained-inadequate	0,417	0,159	0,022	0,145	0,071	0,095
infinite	0,999	1,098	0	0,999	0,159	0,206

Table S2. Numerical values for quantitative comparison of external validation metrics across variable scenarios with mock behavioral data, related to Figure 6.
