# Supplementary material for: Evaluation of unsupervised learning algorithms for the classification of behavior from pose estimation data
Source: Patterns (N Y). 2025 Apr 22;6(5):101237. doi: 10.1016/j.patter.2025.101237 (PMC12142628; doi:10.1016/j.patter.2025.101237)
Supplement: Document S2. Article plus supplemental information [file mmc4.pdf]

Article

Patterns

Evaluation of unsupervised learning algorithms for the classification of behavior from pose estimation data

Highlights

- Unsupervised learning allows to cluster pose-estimation data into behavioral motifs
- Available tools differ in both architecture and output characteristics
- The overall performance of the tested methods was comparable
- Guidance on the use of available tools in specific experimental scenarios

Authors

Jakub Mlost, Rame Dawli, Xuan Liu, Ana Rita Costa, Iskra Pollak Dorocic

Correspondence

jakub.mlost@scilifelab.se (J.M.),
iskra.pollak@scilifelab.se (I.P.D.)

In brief

This study explores recent advances in unsupervised learning algorithms for classifying animal behavior, addressing limitations of traditional behavioral tests and pose-estimation tools. By comparing four algorithms—B-SOiD, BFA, VAME, and Keypoint-MoSeq—the authors provide insights into their clustering efficiency, methodological differences, and biological relevance. These findings offer critical guidance for researchers seeking to apply machine learning in behavioral neuroscience, paving the way for unbiased, scalable analysis of complex behaviors and advancing understanding of the neural mechanisms underlying naturalistic behavior.

Mlost et al., 2025, Patterns *6*, 101237
May 9, 2025 © 2025 The Author(s). Published by Elsevier Inc.
https://doi.org/10.1016/j.patter.2025.101237

CellPress

Patterns

CellPress
OPEN ACCESS

Article

Evaluation of unsupervised learning algorithms for the classification of behavior from pose estimation data

Jakub Mlost,[1,*] Rame Dawli,[1] Xuan Liu,[1] Ana Rita Costa,[1] and Iskra Pollak Dorocic[1,2,*]
[1]Science for Life Laboratory, Department of Biochemistry and Biophysics, Stockholm University, Stockholm, Sweden
[2]Lead contact
*Correspondence: jakub.mlost@scilifelab.se (J.M.), iskra.pollak@scilifelab.se (I.P.D.)
https://doi.org/10.1016/j.patter.2025.101237

THE BIGGER PICTURE Understanding animal behavior is crucial for decoding how the brain works, modeling neurological disorders, and assessing treatments. Recent technological advances have opened new opportunities to capture and classify animal movements and behaviors with unprecedented accuracy. By bridging the gap between movement tracking and behavior classification, these tools promise to revolutionize neuroscience by providing scalable, unbiased methods for behavioral analysis and enabling researchers to uncover previously unknown patterns in animal behavior. However, these tools are new and largely unvalidated, with no objective metrics available to determine the best tool for a given task. This study aims to provide a better understanding of these algorithms, offering qualitative and quantitative comparisons to aid researchers in selecting the most suitable tool for their specific research needs.

SUMMARY

Analyzing animal behavior is crucial for decoding brain function, modeling neurological disorders, and assessing therapeutics. Recent advances in pose-estimation tools like DeepLabCut and SLEAP have revolutionized behavioral analysis by enabling precise tracking of animal body movements. However, these tools do not automate behavioral classification. Unsupervised learning algorithms address this gap by identifying clusters of recurring behavioral motifs from pose-tracking data without requiring pre-labeled datasets, reducing observer bias and uncovering novel patterns. This study compares four recent unsupervised learning algorithms—B-SOiD, BFA, VAME, and Keypoint-MoSeq—analyzing their methodological approaches, clustering efficiency, and ability to produce meaningful behavioral classifications. By offering a qualitative and quantitative evaluation, this paper aims to aid researchers in selecting the most suitable tool for their specific research needs.

INTRODUCTION

Behavioral tests are fundamental to modern neuroscience research. By correlating animal behavior with brain activity or manipulations, researchers can infer the functional roles of specific brain regions, circuits, cell types, and genes.[1–3] Behavioral tests are also used to validate models of neurological disorders such as Parkinson's or Alzheimer's disease by uncovering motor or memory impairment.[4] Furthermore, the same tests allow the assessment of the efficacy of novel drugs.[5] However, traditional behavioral tests typically isolate singular behavioral components under controlled laboratory conditions. For instance, novel object interaction duration is used as a proxy for memory function or locomotor activity in an open field test as an indicator of motor impairment.[6] These measurements are inherently constrained and do not give insight into the complex nature of animal behavior.[7]

Recent developments in pose-estimation tools like DeepLabCut[8,9] and SLEAP[10] have transformed behavioral analysis by enabling precise tracking of animal body positions in video recordings with previously unattainable accuracy. DeepLabCut and SLEAP utilize supervised machine learning methods requiring minimal training data to generate reliable tracking of multiple body parts throughout experimental recordings. The final output of these tools is a data frame providing the X and Y coordinates for each tracked anatomical marker (i.e., keypoint) across all video frames (Figure 1). While these tools excel at movement tracking, they do not address the subsequent challenge of automated behavioral classification.

© 2025 The Author(s). Published by Elsevier Inc.
This is an open access article under the CC BY license (http://creativecommons.org/licenses/by/4.0/).

1 Video frames (Input for DLC)

2 Pose-estimation via DLC

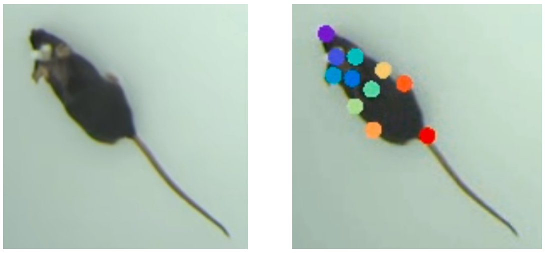

3 (X,Y) cordinates for all bodyparts (Input for all unsupervised algorithms)

Frame	Nose (x)	Nose (y)	Nose (Likelihood)	Headcenter (x)	Headcenter (y)	Headcenter (Likelihood)
1	514,343251	60,054844	0,9889	500,448485	70,592551	0,9626
2	514,232532	59,568995	0,9555	500,562222	69,658411	0,9587
3	514,112333	58,845510	0,9989	500,595621	68,982000	0,9987
4	513,985222	58,843003	0,9822	499,651582	68,595123	0,9824
5	512,542399	59,451236	0,9365	498,622152	69,323211	0,9366
6	511,999845	60,255564	0,9442	497,642295	70,528477	0,9444

Figure 1. DeepLabCut workflow
The input is a raw video of animal behavior, in this case, movement in an open field (1). Example frames were extracted from the video and annotated by a researcher. A model based on recurrent neural network was trained on the manual labeling, and the labeling was extrapolated to new videos (2). We employed 11 keypoints in the present study: nose, head-center, neck, ear left, ear right, body-center, body-center left, body-center right, hip left, hip right, and tailbase (2). Snippet of the DeepLabCut output data from a 650 × 600-pixel video (3). Final output is a data table containing the X and Y coordinates of each labeled body part in each frame, as well as the likelihood of each assignment (the probability of observing the provided data given the parameters in our model).

Recently, unsupervised learning has emerged as a transformative approach for automatic identification and classification of behavior.[7] Unsupervised learning algorithms can discover patterns and structures within data without requiring prelabeled training examples. In the context of behavioral analysis, these algorithms can automatically identify discrete clusters of data points within pose-tracking data. In machine learning terminology, cluster refers to a group of data points that is distinct from another group of data points based on several different metrics. In the case of behavioral analysis, clusters can be functionally interpreted as distinct behavioral motifs (i.e., recurring patterns of animal behavior based on body position). For the purposes of this paper, the terms “clusters” and “motifs” are used interchangeably to describe these fundamental behavioral units.

Clustering algorithms offer a robust and scalable solution to the challenge of behavior classification, addressing key limitations such as the dependence on extensive labeled datasets. Moreover, by removing observer bias, unsupervised learning has the potential to uncover previously unknown behaviors, and to predict the probability of future behavioral sequences. These technological advances have opened the door to computational neuroethology, aimed at deciphering the structure of naturalistic behavior and its underlying neural mechanisms.[7,11]

This paper analyses the performance of four unsupervised learning algorithms: behavioral segmentation of open field in DeepLabCut (B-SOiD),[12] behavioral flow analysis (BFA),[5] variational animal motion embedding (VAME),[13] and Keypoint-MoSeq.[14] These were selected as the most recent and most commonly used methods in the field with significant differences from each other in how they approach the data. We present a qualitative comparison of these methods, providing detailed methodological descriptions and highlighting their similarities and differences. Next, we perform a quantitative comparison, evaluating the clustering efficiency and cluster characteristics. The goal of this study was to provide a comparative analysis of these algorithms, highlighting their strengths and weaknesses, to guide researchers in selecting the most suitable method for their specific research questions.

RESULTS

Qualitative comparison: Feature space

Data preprocessing and feature engineering are initial and fundamental steps in any data analysis pipeline. This is particularly true in the case of raw pose-estimation data, which represents the X and Y coordinates of each body part in every frame of the video, making up a highly dimensional dataset with thousands of relative, time-dependent data points.

To address this issue, B-SOiD first performs a summation/averaging over 100-ms data points to create a set of features involving inter-point delta position (distance between points), frame-to-frame inter-point delta angle, and frame-to-frame delta position (speed). This feature space is then reduced using uniform manifold approximation and projection (UMAP). Therefore, each B-SOiD data point represents a relative change between points over 100 ms.

A similar feature engineering approach is applied in BFA, where distances, angles, accelerations, and so forth are calculated on a frame-to-frame basis. However, BFA includes more features, adding areas between points as well as proximities to the arena’s border, totaling 41 features. BFA addresses the time-dependent aspect of the data using a rolling time window. This transformation creates a vector for each feature at each time point, overall containing data points from surrounding 30 frames around the time point of interest. As a result, each data point (frame) has 1,271 features (41 features × 31 frames). Importantly, adding new features to BFA is relatively straightforward and allows the possibility of including different environmental factors present in more sophisticated behavioral tests.

In contrast, VAME and Keypoint-MoSeq address the relativity of the data points in a different way, by first performing egocentric alignment of the body parts. VAME creates samples containing sequential data for each body part over a predefined time window (default = 30 frames) that are then fed as input for training the variational autoencoder. The purpose of a variational autoencoder is to perform non-linear dimensionality reduction using a bidirectional recurrent neural network that allows retention of the sequential nature of raw data. To simplify, VAME

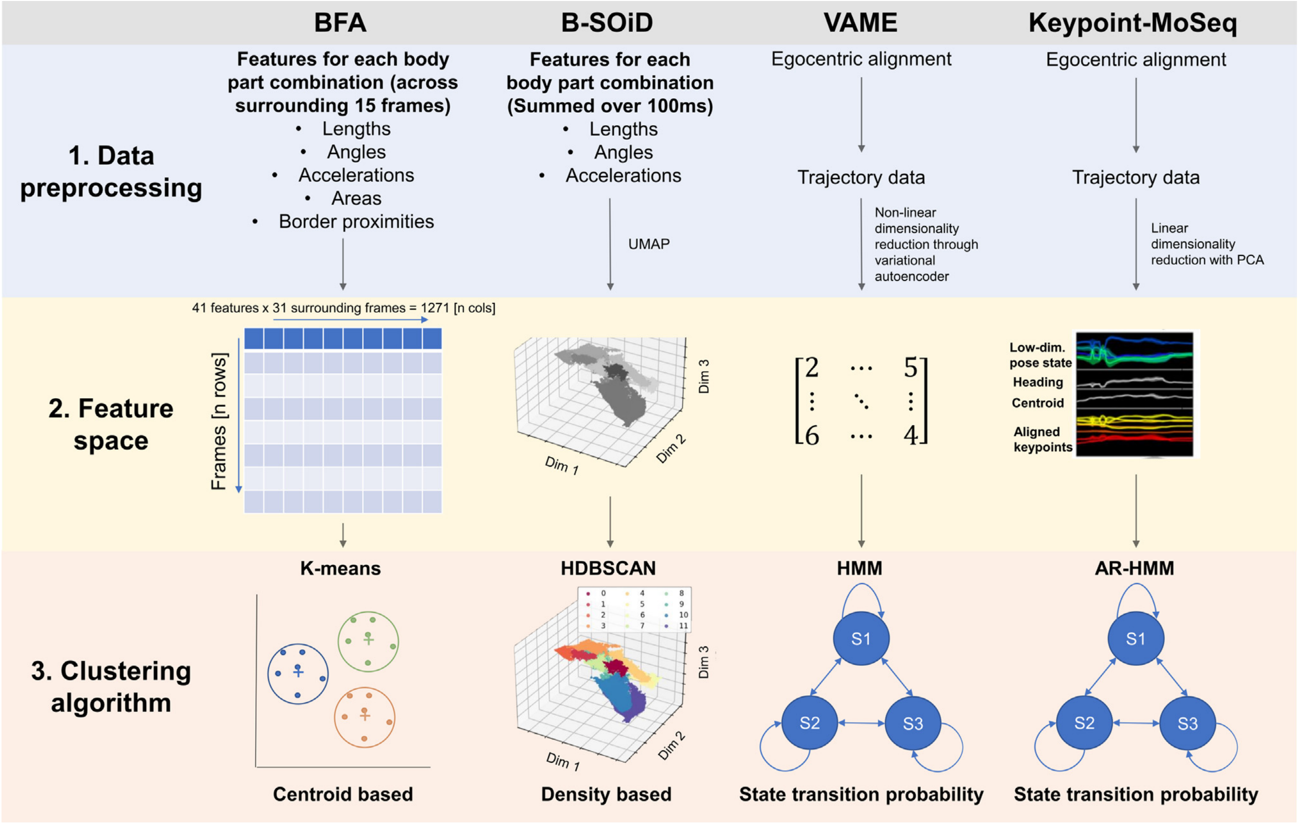

Figure 2. Overview of unsupervised learning algorithms for behavioral classification

Summary of unsupervised learning algorithms for behavioral classification, including comparisons of data preprocessing, feature space, and clustering algorithms.

creates a latent space of sequential data that is then fed to the clustering algorithm. Keypoint-MoSeq instead applies a simpler linear approach to perform dimensionality reduction using principal component analysis (PCA). Both VAME and Keypoint-MoSeq address noise, such as implausible sudden movement of the body parts, during the feature engineering step. Specifically, VAME implements a Savitzky-Golay filter to smooth the time series, as well as thresholding based on the interquartile range, to robustly cut out outliers within the signal. Keypoint-MoSeq addresses noise in the pose estimation data by switching the linear dynamical system model that separates signal from noise by considering each keypoint's data within a broader context of pose dynamics, location, and heading information. When a keypoint shows an implausible sudden movement, the model attributes this to noise rather than real behavior, thereby maintaining a smoother and more accurate pose trajectory. We can therefore arrange the pipelines by the amount of data transformation such as BFA < Keypoint-MoSeq < B-SOID < VAME (Figure 2).

In general, the more transformed the input data is, the harder it can be to understand the relationship between clustering and the input data. This can raise unexpected issues. For example, UMAP dimensionality reduction used by B-SOiD may cause individual data points to look more similar to one another than they really are or, as in case of VAME, make it very hard to extrapolate clustering to new datasets (explained in depth in the section "label transfer"). It is worth noting that from among all available tools, only BFA allows users to freely add new features into input data. While it is also possible to add new features as keypoints into the other tools, this could produce unforeseen and suboptimal outcomes.

Clustering algorithm

Clustering algorithms identify patterns in data by grouping similar data points into clusters, which in the case of behavioral classification can represent behavioral motifs. In B-SOiD, a hierarchical clustering method, HDBSCAN, is used to extract dense regions separated by sparse regions. Some of the advantages of HDBSCAN clustering are automatic cluster discovery: HDBSCAN automatically determines the number of clusters in the dataset without requiring a prior specification; handling cluster shapes: density-based clustering methods can discover clusters of arbitrary shapes, which is not possible with other clustering algorithms like K-means; handling noise: density-based clustering algorithms can automatically identify noise or outliers, as these are typically far from high-density areas.

In contrast, BFA uses K-means clustering, which groups data around centroids using Euclidean distance. While simpler, K-means has limitations for behavioral classification. For instance, determining the optimal number of clusters is challenging in complex datasets, and K-means struggles with

non-centroid-shaped data, such as time series keypoints (see Figure S1 for an example).

VAME and Keypoint-MoSeq take a different approach by using the hidden Markovian model (HMM) to infer changes in hidden states (i.e., behavioral motifs) from observed variables (keypoints in latent space). In an autoregressive HMM (AR-HMM) used by Keypoint-MoSeq, the current observation is modeled as a linear combination of previous observations. This adds a temporal dependency to the observations, which is crucial for capturing the dynamics of time series data, such as animal behavior.

A key difference between VAME and Keypoint-MoSeq lies in how the number of motifs is determined. In VAME, the user must predefine the number of motifs, similar to K-means. Conversely, Keypoint-MoSeq employs a sticky hierarchical Dirichlet process, which infers the number of hidden states directly from the data, making it more adaptive.

To summarize, B-SOiD and Keypoint-MoSeq stand out for their ability to optimize the number of biologically meaningful clusters directly from the data. In contrast, VAME and BFA require additional steps to determine the number of clusters, which can lead to over- or under-representation of true behavioral motifs. Furthermore, B-SOiD and BFA primarily segment behavioral recordings into distinct motifs based on an animal's position and movement in the current as well as surrounding frames, resulting in detection of discrete behavioral motifs such as walking, sniffing, or turning. However, VAME and Keypoint-MoSeq identify clusters based on the sequences of positions, resulting in the detection of behavioral motifs such as walking and sniffing or walking and turning (see the supplemental videos in data and code availability for examples).

Label transfer

A major limitation of clustering algorithms in unsupervised learning models is their dependence on the input data, which often leads to a lack of reproducibility. This means that the same algorithm may produce varying clusters when applied to different batches of data (e.g., distinct sets of behavioral videos). Label transfer refers to the process of applying cluster identities learned from one dataset to another, typically using a supervised classifier trained on the initial dataset. This approach enables consistency in cluster assignments across datasets. The present section discusses methods employed in each workflow to address this challenge. We mainly focus on aspects such as out-of-the-box functionality and robustness, which is the ability to perform well on new and unseen data.

In B-SOiD and BFA, label transfer is achieved by leveraging distinct classification strategies. B-SOiD employs a random forest classifier trained on feature space data prior to dimensionality reduction, allowing it to label new data effectively, provided the new dataset is not substantially different from the original. In contrast, BFA uses a sequential neural network, which generally excels at generalizing to new data, offering increased flexibility in more varied datasets.

Keypoint-MoSeq provides the most robust framework for label transfer. Its AR-HMM predicts transitions between hidden states (behavioral motifs) based on raw data processed through linear PCA. Once trained, the AR-HMM can seamlessly apply the learned motifs to new datasets that undergo the same PCA transformation, ensuring consistent and reproducible clustering.

In contrast, VAME faces significant limitations in label transfer due to its reliance on variational autoencoders, which non-linearly transform raw data into a latent space. This process generates latent representations that are dataset specific, preventing it from transferring labels from one dataset to another using the original HMM model.

In summary, Keypoint-MoSeq stands out as the most user-friendly and robust tool for label transfer, as it works out-of-the-box for any new dataset. BFA follows, with its sequential neural network, although it may require fine-tuning to achieve optimal performance across different datasets. B-SOiD, with its random forest classifier, is only effective when datasets remain similar to the training set, but fails to produce adequate results if the new dataset is sufficiently different from the original data. Unfortunately, VAME's lack of a native label transfer mechanism and the latent nature of its feature space pose a significant limitation to its reproducibility.

Data visualization and motif examples

Data visualization is an essential and often troublesome part of scientific research, especially in the context of big data. All four packages offer tools to render video examples of discovered motifs that differ in subtleties discussed below. B-SOiD and VAME provide separate videos for each behavioral motif, where separate examples are sequentially displayed. B-SOiD provides some adjustments to render examples of the desired minimum length. Keypoint-MoSeq renders the videos, where multiple examples are shown at the same time and aligned to the beginning of the motif. However, looking at multiple examples at once is sometimes confusing and makes it harder to interpret the biological meaning of the motif. BFA provides the most flexible tool for video rendering, but it comes at the price of ease of use due to difficulties in integrating and implementing video editing ffmpeg library with R studio code.

When it comes to data visualization, B-SOiD offers only a UMAP representation of the feature space (Figure 3A), and simple transition graphs as well as limb trajectories at a specific time point. VAME offers a tool to visualize community hierarchical graphs presenting similarity between clusters. BFA offers a very interesting toolset for data visualization, including the ability to visualize how behavioral motifs produced by different clustering algorithms align to one another or to the ground truth labels (Figure 3B) (e.g., allowing the user to visualize what behavioral motifs correspond to the beginning or ending of a rearing) (Figure 3C). BFA can also visualize various transition graphs that either show down- or up-regulation of the transitions between behavioral motifs (Figure 3D).

Basic data analysis of behavioral motifs produced by unsupervised learning algorithms can focus on either the duration of the motifs or the transitions between them. However, this approach suffers from a dimensionality curse and multiple comparison problems. Thus, researchers in the field need to provide a novel approach to overcome these problems. Most noteworthy is the behavior flow fingerprinting (BFF) tool included in BFA, which allows visualization of overall differences in behavioral phenotype based on transition matrices in different treatment groups (Figure 3E). To calculate BFF, the transition matrix of behavioral motifs, representing the individual's unique behavioral flow, is subjected to dimensionality reduction techniques, creating a single

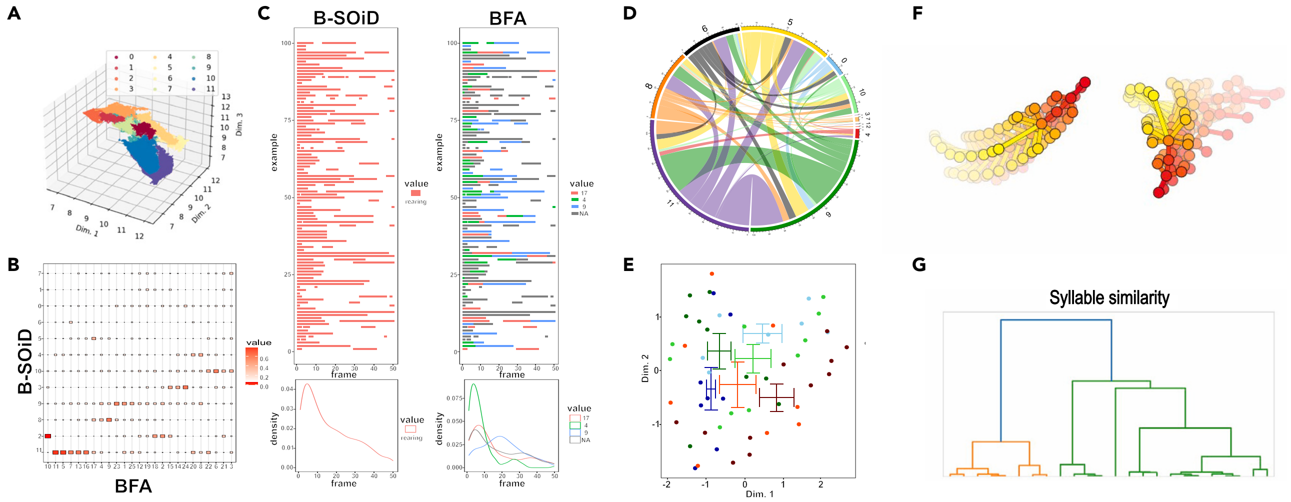

Figure 3. Visualization examples produced by different pipelines

B-SOiD allows the user to assess cluster separation in a UMAP plot (A). BFA allows the user to compare clustering results produced with different methods as a confusion matrix (B) or to visualize temporal dynamics in a side-by-side comparison (C). For example, a rearing cluster produced by B-SOiD (left side, C) is visualized against clusters annotated by BFA (right side, C), where one can see that BFA cluster 4 corresponds to the beginning of the rearing behavior and cluster 9 to the end of the rearing behavior (right bottom, C). BFA also provides tools to visualize transition graphs of all transitions, as well as only those that are either up- or down-regulated (D). BFA provides tools for dimensionality reduction pipeline to visualize global differences in behavioral phenotype (E). Keypoint-MoSeq allows visualization of the patterns in behavioral motifs (F), as well as the similarity between different clusters (G).

data point in a lower-dimensional space. This allows researchers to visualize subgroups of animals based on their behavioral responses and, for instance, compare effects of different drugs or observe dose-response effects at the group level. In particular, one could use BFF to distinguish between responders and non-responders to experimental treatments. In stress research, for example, BFF can help identify animals susceptible to stress-induced behavioral changes versus resilient animals.

Finally, Keypoint-MoSeq provides probably the most powerful tool as it visualizes the skeleton of keypoints in different behavioral motifs in both a stable (as a PDF) (Figure 3F) and dynamic way (as a GIF), which makes interpretation of the clusters easier. It also produces a syllable dendrogram that represents the similarity in trajectory between each motif (Figure 3G).

Technical aspects (perspective, frequency, keypoints, environment)

The above-mentioned aspects focus on major architectural differences between methods; however, there are also minor technical differences. For example, the type of data used for method development can change the quality of clustering if used with data with other characteristics.

There are two main technical differences in behavioral recording acquisition that can impact the clustering algorithm: perspective (top-down vs. bottom-up) and video frequency. Standard recording setup employs a top-down perspective of the animal and video frequency ranging from 25 to 30 Hz, as used by both BFA and Keypoint-MoSeq. B-SOiD and VAME were instead developed on a more advanced setup involving bottom-up recordings in 60 Hz. To improve the signal-to-noise ratio, B-SOiD downsamples all input to non-overlapping 10-fps (100 ms) windows, and then either sums (displacement, angular change) or averages (distance) over all 10-fps samples.

However, VAME authors recommend feeding at least 20 data points for the recurrent neural network to work properly, which, in the case of a 60-Hz recording equals to 0.3 s of the recording, while in a more standard 25-Hz recording, the algorithm uses almost 1 s to learn an embedding from the kinematic trajectory of the moving animal.

Another variable is that bottom-up recording may contain more kinematic information about the animal's behavior that can then be used by the clustering algorithm, compared to more standard top-down recording. It is worth noting that Keypoint-MoSeq has been presented as working on many different recording setups, using overhead or bottom-up camera angles, with two- or three-dimensional (3D) keypoints, and in both mice and rats.[14] B-SOiD has also been successfully used in top-down, low-frequency setting,[1,12] as well as side view in pain studies.[15] Considering the above, Keypoint-MoSeq presents itself as the most robust method for different recording setups, while B-SOiD has also been shown to work with multiple setups. BFA and VAME appear to be more rigid. BFA requires specific keypoints to work properly (see next paragraph), while VAME can underperform in setups with a slower camera and top-down recordings, as in the present study.

In addition to the video perspective, it is important to consider the choice and number of keypoints, as this can significantly impact the quality of the model. For instance, VAME and B-SOiD were initially developed using a lower number of keypoints (six), which was partly due to their reliance on a bottom-up perspective that provided rich kinematic information even with fewer tracked points.[12,13] In contrast, Keypoint-MoSeq was tested against a range of keypoint configurations, demonstrating its robustness under varying conditions (different perspectives, including 3D model).[14] While B-SOiD, VAME, and Keypoint-MoSeq are agnostic to the specific body parts used

Table 1. Summary of different technical aspects of each unsupervised learning algorithm designed for the discovery of distinct behavioral motifs

Method	Noise handling	Environmental features	Cluster types	Cluster number optimization	Label transfer	Visualization tools
B-SOID	significant	X	non-sequential	✓	✓	simple
BFA	X	✓	non-sequential	X	✓	advanced
VAME	moderate	X	sequential	X	X	simple
Keypoint-MoSeq	significant	possible	sequential	✓	✓	advanced

as input, BFA imposes a stricter requirement: it expects exactly the same keypoints as those used in the original paper.[5] This constraint limits its applicability primarily to rodents and top-down view, whereas the other methods can be more flexibly applied across different model organisms with different recording setups.

One application is to use these algorithms to study animal interactions with the environment. Only BFA employs parameters such as distance to the border of the arena, and its architecture allows for easy manipulation and addition of the input variables, such as distance from the object of interests. B-SOiD and VAME do not account for environmental factors, and including them as additional keypoints might cause some unexpected behavior in the algorithm, especially in case of VAME, which uses egocentric alignment. Although not discussed in the original paper, the Keypoint-MoSeq authors developed a "location-aware" model that allows the animal's location and heading to depend on the current motif (described in depth in the Keypoint-MoSeq documentation website). This model, however, remains experimental and may produce some unexpected behavior. A summary of the different technical aspects of each method can be found in Table 1.

Finally, it is important to mention factors relevant to user experience. In our case, we did not experience any difficulties with the installation of the tools. Most of the packages, namely B-SOiD, VAME, and Keypoint-MoSeq, were created using Python language, while BFA was created in R. B-SOiD provides a graphical user interface that allows it to be used by users with no coding experience.

Limitations of qualitative comparison

All of the evaluated methods rely on pose-estimation data, which can be obtained using tools such as DeepLabCut or SLEAP. These programs leverage deep neural networks, renowned for their broad applicability and robustness. Consequently, pose-estimation data can be collected under virtually any condition, including various species, variable and natural settings, and multi-animal recordings. However, it is important to note that these unsupervised learning algorithms were primarily developed for rodents, the most common model organisms in neuroscientific research. In theory, some of these tools should also be applicable to other species; for example, Keypoint-MoSeq has been demonstrated with fruit fly recordings.[14] Nonetheless, assessing their usability in non-rodent species is beyond the scope of this paper.

Social behavior is another key area of interest in neuroscience. Although the presented methods do not directly support pose estimation for multiple animals, they can process data from a single animal within a multi-animal environment, potentially revealing distinct social behavior motifs. For studies focused on inter-animal interactions, only BFA currently offers an open environment that allows for the addition of features related to such interactions (e.g., measuring the distance between familiar and novel animals). While adding an extra keypoint that would mark other animals in the video in other tools is possible, this approach may yield unexpected results and also falls outside the scope of this paper. Other tools employing a supervised (such as SimBA or DLCanalyzer, among others; see Isik and Unal[18] for review)[16–18] or semi-supervised[19,20] approach might be better suited for addressing questions related to social behavior.

Quantitative comparison: Motif characteristics

One of the most prominent differences between the algorithms is the motif duration and the number of detected clusters, both of which significantly influence the biological interpretation of the behavioral clusters. As mentioned above, VAME and BFA require the user to choose the cluster number *a priori* and with some optimization. In accordance with the authors' recommendations, we found the optimal number of clusters to be 25 for BFA and 40 for VAME. In contrast, B-SOiD and Keypoint-MoSeq automatically optimize the number of clusters. In our experimental videos, B-SOiD detected 12 clusters, while Keypoint-MoSeq detected 24 unique clusters. In terms of the number of clusters, methods were ranked as follows: B-SOiD < Keypoint-MoSeq < BFA < VAME.

B-SOiD and VAME produced the shortest motifs, with median durations of 0.44 and 0.46 s, respectively (Figures 4A and 4B). BFA and Keypoint-MoSeq produced longer motifs, with median durations of 0.68 and 0.72 s (Figures 4C and 4D). We checked for statistical differences using Kruskal-Wallis with Dunn's post hoc test with Holm's correction and all the comparisons were statistically significant, supporting differences in the medians. It is important to note that users can largely adjust motif duration in Keypoint-MoSeq and VAME as a hyperparameter, which is not explicitly possible in B-SOiD and BFA, but it could eventually be adjusted to one's preferences by changing a given number of frames per second. Importantly, Keypoint-MoSeq allows one to adjust the kappa value, which can affect both the number of clusters and the median duration of behavioral motifs (i.e., longer behavioral motifs result in lower number of clusters).

Consistency across different samples

To establish consistency of clustering across samples, we performed statistical analysis to compare medians of each

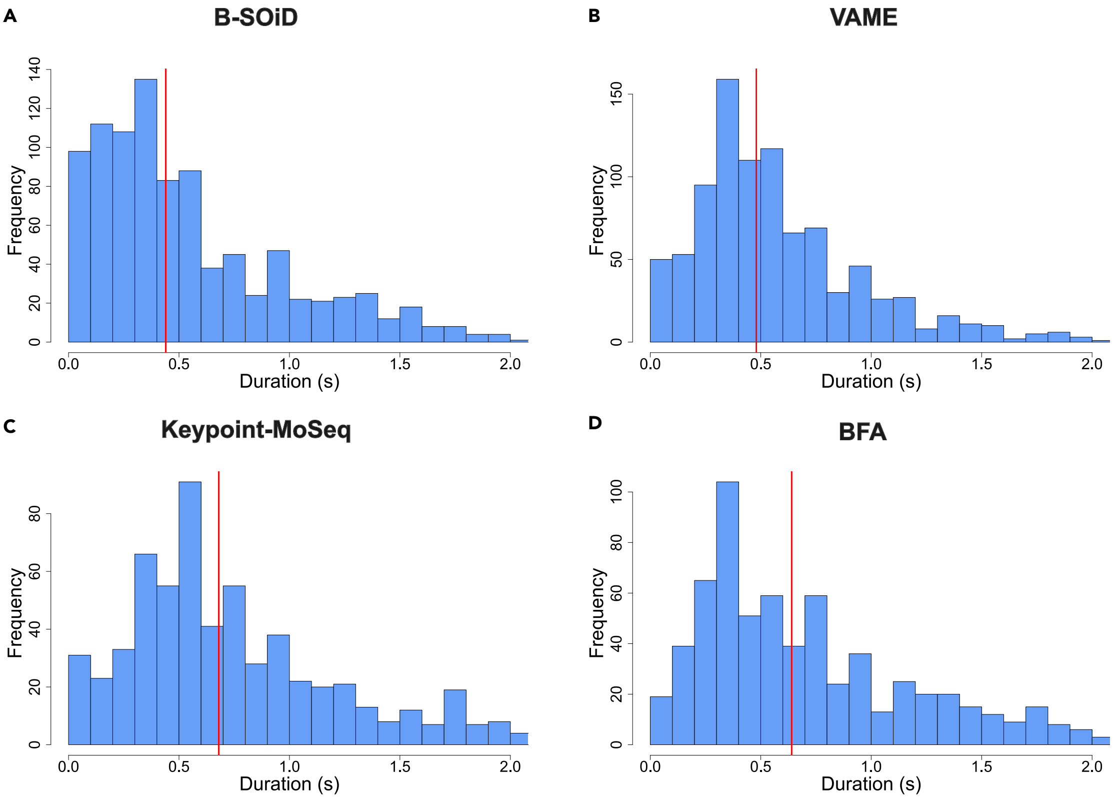

Figure 4. Duration of behavioral motifs
Frequency of behavioral motifs with different lengths for B-SOiD (A), VAME (B), Keypoint-MoSeq (C), and BFA (D). Red lines denote median.

behavioral motif across 10-min videos of three wild-type naive mice. We did not find any differences in median duration of any behavioral motif produced by B-SOiD (Figure 5A), suggesting a very consistent behavioral phenotype detected by this method. With VAME and Keypoint-MoSeq, we found differences in median duration of behavioral motif 28 (Figure 5B) and 1 (Figure 5C), respectively. However, we were not able to conduct statistical analysis due to the low number of occurrences (<3) for as many as 9 behavioral motifs produced by VAME (motifs 1, 14, 15, 21, 24, 26, 30, 35, and 38). Keypoint-MoSeq produced only one behavioral motif that occurred fewer than three times (motif 4). For BFA, we did not detect any differences in median duration of behavioral motifs; however, we were unable to analyze motifs 10 and 15 due to insufficient data points. The number of occurrences of each motif can be found in Table S3 and the time and frequency of each behavioral motif produced by different methods can be found in Figures S2–S5.

These results present B-SOiD as the most consistent method for behavioral segmentation at the cost of resolution (the lowest number of behavioral motifs detected). Significant differences in the median duration of some behavioral motifs between wild-type naive animals can suggest their increased sensitivity for detection of more subtle behavioral motifs that are uniquely expressed between animals. However, that aspect comes at a high price for VAME clustering, which produces too many clusters that are often not present in different animals. Perhaps lowering the number of clusters could increase the performance of VAME clustering, although one would need to develop a novel approach to assess the right number of clusters, which is a difficult task (see further sections on validity measures). In this evaluation, BFA exhibits intermediate performance between consistency and resolution.

Validity measure

In unsupervised learning, ensuring the validity of identified behavioral clusters is critical for making accurate inferences about animal behavior. While data-driven approaches offer the potential for unbiased discovery, the absence of ground truth labels and the complexity of animal behavior data pose significant challenges to assessing the validity of the clustering. Developing robust validity measures that account for these challenges will be crucial for advancing the field of unsupervised learning in behavioral neuroscience and ensuring the reproducibility and reliability of findings.

In the present work, we have applied some of the gold standard methods used to validate the clustering. These methods can be generally divided into two types: internal or external validation measures. Internal validation assesses the cohesiveness and separation of clusters or patterns based solely on the dataset and clustering results, using metrics such as silhouette scores or the Davies-Bouldin index. In contrast, external validation compares the clustering results to a known ground truth or external labels (if available) using metrics like adjusted Rand

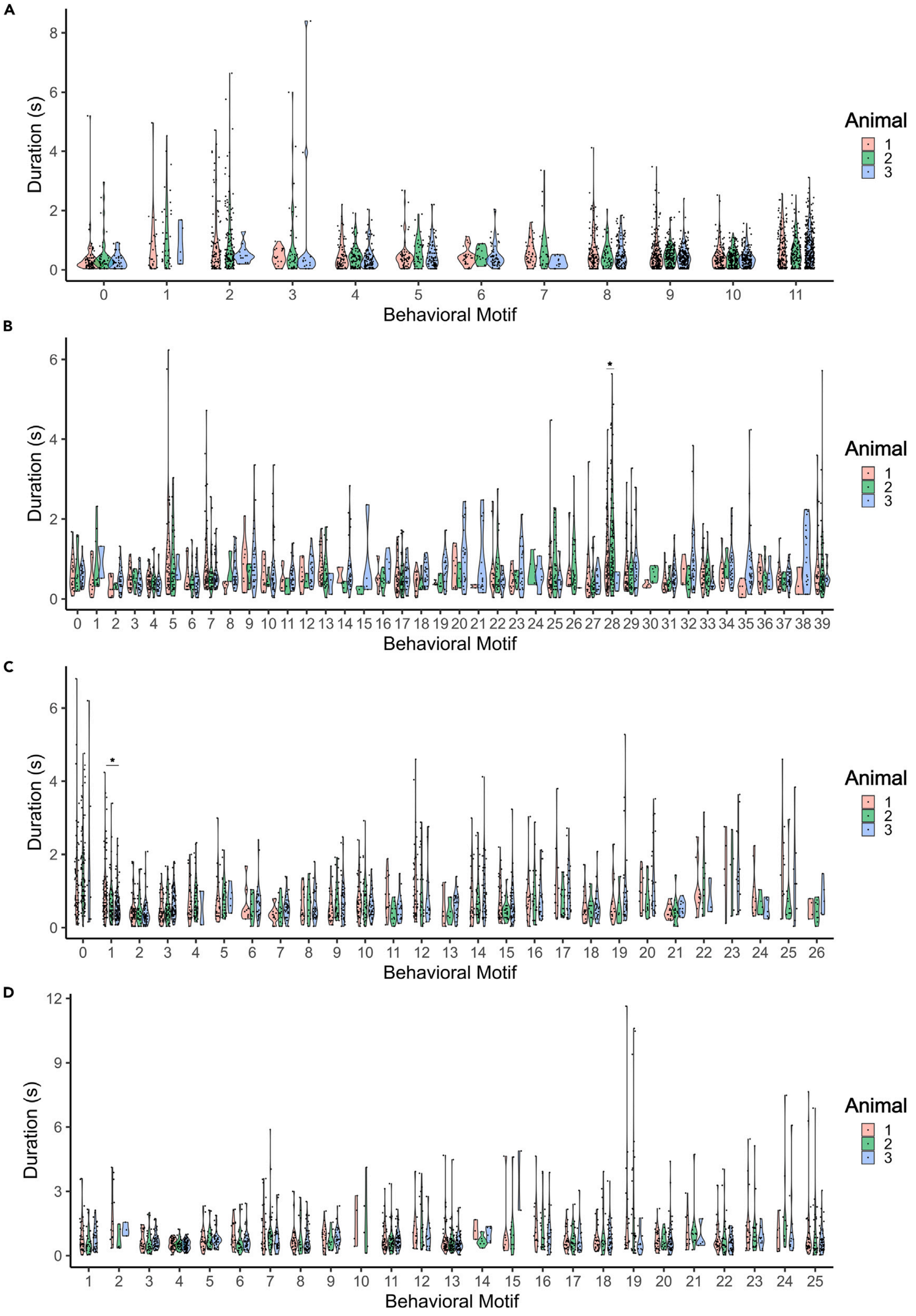

(legend on next page)

index (ARI) or normalized mutual information, providing an objective benchmark for evaluation.

It is important to emphasize that all clustering validity measures are biased toward specific metrics and will likely prefer one clustering method over another. For the current comparison, we chose silhouette score and modified ARI (MARI) as the most reliable tools to assess the clustering efficacy of the behavioral data.

Silhouette score

We first employ silhouette score, which is a gold standard internal clustering measure (i.e., it gives us information on how well the clusters of data points are separated from one another).[21] The silhouette value is a measure of how similar an object is to its own cluster (cohesion) compared to other clusters (separation). The silhouette ranges from −1 to +1, where a high value indicates that the object is well matched to its own cluster and poorly matched to neighboring clusters. The silhouette score is specialized for measuring cluster quality when the clusters are convex shaped and may not perform well if the data clusters have irregular shapes or are of varying sizes, as in the case of time series keypoint data.

We have performed silhouette score calculations for each method using their respective feature data. Not surprisingly, we observed the best separation of data points with B-SOiD (Figure 6A). VAME and BFA provided similar mediocre scores, while Keypoint-MoSeq significantly underperformed in this scoring method (Figure 6A; Table S1).

The silhouette score analysis highlights a key distinction between the clustering approaches employed by the different methods. B-SOiD, which focuses on identifying clusters based on feature space separation, achieves the highest silhouette score, indicating a clear separation between clusters. This suggests that for analyses prioritizing well-defined and distinct behavioral motifs, B-SOiD might be the most suitable choice. In contrast, VAME and Keypoint-MoSeq, which utilize HMM, prioritize patterns in data sequences. Consequently, their lower silhouette scores are expected, as they do not primarily aim for feature space separation. These methods might be more appropriate when the research focus lies in capturing the temporal dynamics and flow of behavior rather than identifying discrete behavioral categories. BFA occupies a middle ground, exhibiting a moderate silhouette score. Although the silhouette score is supposed to be positively biased specifically toward centroid-based K-means clustering results, the very high dimensionality of the feature space used by BFA could significantly lower it. Ultimately, it is challenging to assess the clustering efficacy of algorithms with such different architectures and data assumptions using strict internal validation measures such as the silhouette score.

MARI

To overcome the limitations of internal validity measures, we performed an additional comparison of clustering against manual labeling of the data. As the clusters obtained by the unsupervised learning algorithms differ in their characteristics, as well as the manual labeling itself being slightly different from what is detected by the unsupervised classification methods, we decided to employ the MARI to assess the clustering efficiency.[22]

Other methods rely on a more direct comparison between ground truth and clustering, while Rand index (RI), a fundamental building block of MARI, is based on a combinatorial approach. That is, each pair of points can be (1) clustered together in both clusterings, (2) clustered separately in both clusterings, (3) clustered together in the hypothesized but not the target clustering, or (4) clustered together in the target but not in the hypothesized clustering. Based on these four values, RI measures the similarity between clustering and ground truth by counting pairs of points that are either in the same or different clusters in both classifications. RI ranges from 0 to 1, and the value can be interpreted as the probability that a pair of points are clustered similarly (together or separately), where 1 indicates that the two data clusterings are exactly the same. Adjusting corrects for a chance to achieve a high score due to the high number of clusters, and the modified version of the algorithm makes it more robust and less susceptible to the violation of the assumptions.[22,23]

Ideal external validation measurement for the purpose of behavioral analysis should give a high score even when the clustering solution is different from the ground truth labeling (i.e., finding more behavioral motifs than is annotated by a human observer). However, the score should decrease if the clustering is redundant and non-consistent (i.e., the number of clusters produced by the algorithm is high, but the clusters are not consistently assigned to the same behavioral motifs observed by a researcher). To validate the appropriateness of MARI as our evaluation metric, we conducted a comparative analysis of available external validation metrics using simulated data that resembled actual behavioral patterns (see methods for details). In this mock data, we assumed that three behavioral motifs assigned by human observers such as "walking and sniffing" and "walking and turning" can be split by unsupervised learning algorithm into either four clusters or, in a fine-grained variant, into 10 different clusters. With these two variants we created a mock clustering solution that would represent 100% (optimal), 75% (suboptimal), or 50% (inadequate) of the desired clustering accuracy.

The comparative results are presented in Figure S6 and Table S2. Our analysis revealed that all external validation algorithms tested, with the exceptions of MARI and purity, assigned higher scores to inadequate solutions containing excessive clusters (fine-grained-inadequate, 50% accuracy) than to more appropriate solutions with fewer clusters (suboptimal, 75% accuracy). Furthermore, these metrics are unbounded, resulting in scores that approach their maximum values as the number of clusters approaches the number of data points (illustrated by the infinite bar in Figure S6). To further assess metric reliability, we distributed 1,000 data points randomly across

Figure 5. Durations of behavioral motifs across samples

Violin plots showing durations of the different behavioral motifs detected by B-SOiD (A), VAME (B), Keypoint-Moseq (C), and BFA (D). Each motif is divided into three colors representing unique 10-min recordings of naive wild-type mice. Red represents sample 1, green represents sample 2, and blue represents sample 3. *Statistically significant difference ($p < 0.05$) detected by Kruskal-Wallis and Dunn's post-hoc test.

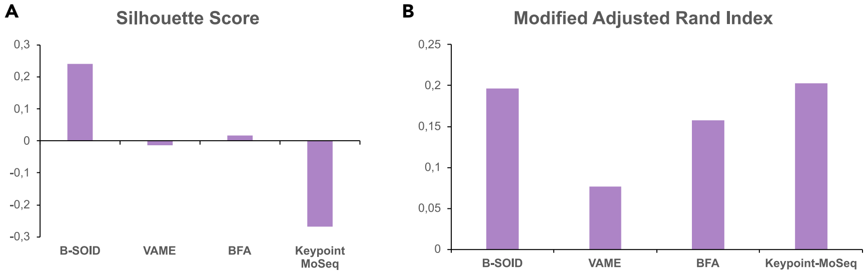

Figure 6. Internal and external validation measures for different clustering algorithms

Silhouette index is an internal validation measurement describing the separation of data points between clusters. (A) MARI is an external validation measurement that describes the similarity between manual labeling and clustering solutions. (B) Positive values denote the best clustering efficacy. Exact values are presented in Table S1.

groups and observed that nearly all metrics were influenced by cluster quantity, with MARI being the notable exception (Figure S7). Although our findings suggested that MARI might favor solutions with fewer clusters, in more realistic scenarios where approximately 75% of data points were correctly assigned, MARI appropriately favored higher-resolution solutions. Based on these comprehensive validation tests, we propose MARI as the most reliable metric for comparing algorithmic clustering results against manual labeling of behavioral data.

According to MARI, we observed the highest score with Keypoint-MoSeq and B-SOiD clustering on par, followed by BFA and VAME (Figure 6B; Table S1). The analysis using MARI reveals key insights into the performance of different clustering methods when compared to manual labeling. The relatively high MARI score obtained by Keypoint-MoSeq, followed by B-SOiD, suggests that these methods effectively capture the underlying structure of mouse behavior as perceived by human observers. BFA demonstrated moderate performance, potentially limited by its emphasis on the animal's spatial location during clustering. VAME, with the lowest MARI score, highlights the potential limitations of the method in detecting temporal patterns in behavior that align with human interpretation and labeling, which could be caused by clustering driven by keypoint jittering. For completeness, we also evaluated methods using the purity metric (Figure S8). Keypoint-MoSeq and BFA achieved comparably high scores, while B-SOiD and VAME showed slightly lower but similar performance. Considering this metric's bias toward solutions with higher cluster numbers, the results suggest that B-SOiD, BFA, and Keypoint-MoSeq are as effective in discerning animal behavior as human scoring. However, VAME's consistently lower performance across metrics raises concerns about its effectiveness for behavioral analysis in sub-optimal recording conditions.

Limitations of quantitative comparison

When choosing the best unsupervised learning algorithm, one can also take into consideration properties such as the ability to detect specific behaviors, such as rearing or grooming, or the overall ability of the algorithm to detect changes in the behavioral phenotype following experimental manipulation.

Regarding behavior-specific accuracy measurements, a fundamental challenge exists: each method produces distinctly different clusters or behavioral motifs (as noted in the clustering algorithm section). This makes direct comparison of specific behaviors impossible, as there is no consistent "rearing cluster" across all methods. Some pipelines combine behaviors (e.g., sniffing with walking, or rearing with walking) that others separate. For instance, VAME's clusters 1, 4, 7, and 12 contain rearing behaviors, but only clusters 4 and 12 represent pure rearing (see the supplemental videos in data and code availability). Similarly, B-SOiD's clusters 2 and 8 include rearing, but only cluster 8 is clearly defined as such (see the supplemental videos). Any attempt to select which clusters to include in a behavior-specific analysis would introduce subjective bias, preventing objective comparison.

Regarding sensitivity to detect changes in behavioral phenotype following experimental manipulation, the BFF analysis toolbox from the BFA package allows for this type of analysis. von Ziegler et al. have successfully employed BFF to detect and quantify changes in behavioral phenotype following different pharmacological and behavioral manipulations.[5] One can apply the same analysis to detect and quantify the sensitivity of different unsupervised learning algorithms to detect changes in the behavioral phenotype. However, this should ideally be assessed on a case-by-case basis because, as presented here, each method has its own characteristics that may affect the sensitivity to detect changes in behavioral phenotype in different experimental conditions.

DISCUSSION

Here, we sought to evaluate which unsupervised learning method delivers the most robust and interpretable results for behavioral analysis, with a focus on its applicability in behavioral neuroscience. While we found that the overall performance of the tested methods was comparable, each exhibited unique strengths and weaknesses based on how they process and interpret behavioral data. These differences highlight their suitability for specific experimental contexts and research questions.

For instance, B-SOiD and BFA prioritize clustering data points in the feature space, resulting in sharply defined behavioral motifs that can be interpreted as discrete segments of the behavioral recording. These methods excel at identifying simple, distinct behaviors such as rearing or sniffing. B-SOiD, with its simplicity and reproducibility, is particularly effective for analyzing straightforward behavioral paradigms like open field tests. Conversely, BFA offers greater flexibility by allowing modifications to the feature space to account for environmental factors, making it well suited for complex settings like the elevated plus maze or novel object recognition tasks.

In contrast, VAME and Keypoint-MoSeq emphasize the sequence and structure of movements, producing motifs that capture both simple and complex behaviors as dynamic,

integrated patterns. The architecture of VAME and Keypoint-MoSeq, which leverages HMM, enables them to parse nuanced movement sequences effectively. However, VAME's limitations must be noted: it is sensitive to noise, leading to spurious clusters (e.g., jittering of keypoints), and it lacks a framework to annotate new data with preexisting models, which hinders reproducibility. Some of VAME's underperformance in our study may be attributed to differences in recording setups; our top-down 25-Hz recording contrasts with the bottom-up 60-Hz configuration used by its developers, which may have impacted its efficacy.

Keypoint-MoSeq, by comparison, emerged as the most robust and versatile method that was validated in multiple recording settings. It not only handles noise effectively but also facilitates the discovery of both short-lived behavioral motifs and extended movement sequences through its tunable kappa parameter. Additionally, it offers advanced cluster visualization tools and features, such as the location-aware model, which disables egocentric alignment to accommodate spatially relevant behaviors. While exploring this feature was beyond the scope of our study, it holds promise for future applications in tasks requiring spatial context.

In summary, the choice of method should be guided by the specific demands of the research question. B-SOiD will excel at finding short and easy-to-interpret behavioral motifs, while BFA is well suited for scenarios requiring environmental adaptability. For studies focusing on complex movement sequences, VAME and Keypoint-MoSeq offer unparalleled depth, with the latter providing superior noise handling, flexibility, robustness, and ease of use. One should also note a significant limitation of VAME's inherent deep-learning architecture (i.e., use of autoencoders) that prevents the user from simply transferring labels to new datasets. This in turn drastically reduces reproducibility of the obtained results. Moreover, a more demanding recording setup as well as the lack of clear guidance on cluster number optimization make this method most difficult to implement, which can lead to poor results, as seen in our case with clustering consistency (Figure 5) and comparison against ground truth labeling (Figures 6B and S8). We hope this comparative analysis serves as a resource for researchers in behavioral neuroscience, enabling them to select the most appropriate tool for their experimental needs.

METHODS

Experiments were carried out in adult C57BL/6J (Charles River) male and female mice. Mice were maintained under standard housing conditions with a 12-h light cycle and with *ad libitum* access to food and water. All animal experiments had received approval from the local ethical board, Stockholm's Norra Djurförsöksetiska Nämnd, and were performed in accordance with the European Communities Council Directive 2010/63.

The open field test was conducted in a 40 × 40-cm arena (Ugo Basil), with recordings made from a top-down view using a Basler acA1920-155um camera. Each mouse was recorded for 10 min, and a total of 56 mice were used for analysis. For body part tracking, we used DeepLabCut (version 2.3.3).[8,9] Specifically, we labeled 24 frames taken from 18 videos (432 frames in total, then 95% [410 frames] was used for training), with 11 body part markers (keypoints): nose, head-center, neck, ear left, ear right, body-center, body-center left, body-center right, hip left, hip right, and tailbase. We used a ResNet-50 neural network with default parameters for 200,000 number-of-training iterations. For the final model, test error: 2.87 pixels and train: 2.46 pixels (image size was 650 × 600). This network was then used to analyze videos from similar experimental settings. Values of points with low likelihood (<0.95) and points tracked outside an existence polygon (arena scaled by a factor 1.3) were removed and interpolated using the R package "imputeTS" (version 3.2).

To choose the optimal models from each method, we followed guidelines reported in the original papers. Briefly, for VAME analysis we used default settings for z_dim parameter and set the time window to 15 frames, corresponding to 500 ms of behavior. To determine the number of clusters in VAME, we let the HMM infer 100 motifs and treat motifs that have <1% usage as noise. This mark was around 40–50 motifs. After inspecting the resulting motif videos for both 40 and 50 clusters and to obtain more compact clusters similar to other methods, the optimal cluster size was identified as 40. Once this number is identified, a new HMM model is trained with this number to segment the final motif distribution. For B-SOiD analysis, we used default values for minimum cluster size, which ranged between 0.5% and 1%, and this provided the highest score in random forest accuracy, >0.95. For Keypoint-MoSeq we used kappa = 1e−6, which also yielded a median motif duration of 520 ms. For BFA, we applied a default pipeline that suggested 25 clusters.

To evaluate the performance of the unsupervised classification, we have performed manual labeling of 3,000 frames from 3 mice (1,000 frames each). Videos were annotated with the following behavioral classification labels: turn right, turn left, walk, stand and sniff, unsupported rear, walk and sniff, supported rear, groom, look up and down, and pause. Three experts agreed on the manually labeled behavioral classification in each frame. For the statistical analysis of median duration of behavioral motifs, we employed the Kruskal-Wallis test with Dunn's post hoc analysis and false discovery rate multiple comparison correction. Only *p* adjusted values <0.05 were considered significant.

To validate assumptions underlying external validity measures, we created simulated behavioral data consisting of 24 data points divided into three equal groups of four data points each (Table S4). Each group was assigned a distinct behavioral label ("walking and sniffing," "walking and turning left," or "walking and turning right"), representing coarse manual labeling that groups similar behaviors into motifs. We then created mock clustering solutions with varying degrees of accuracy. In the optimal clustering solution (100% accuracy), two frames from each bin were assigned as "walking," while the remaining two were correctly assigned as "sniffing," "turning left," or "turning right." In the suboptimal solution (75% accuracy), one datapoint from each bin was misassigned to a cluster from another bin. In the inadequate solution (50% accuracy), two frames from each bin were correctly assigned as "walking," while the other two were randomly assigned behaviors from different bins. This approach maintained a consistent number of clusters (four) across solutions.

We also created higher-resolution clustering scenarios where "walking" was divided into four distinct clusters and each remaining behavior into two clusters, resulting in 10 distinct clusters compared to the three ground truth groups. For these higher-resolution scenarios, we similarly created suboptimal and inadequate solutions, achieving 75% and 50% accuracy, respectively.

Each dataframe was replicated 42 times to generate 1,008 data points, reflecting the approximately 1,000 frames per animal in our actual manual labeling dataset. We also included an extreme case ("infinite") where the number of clusters equaled the number of data points minus one. To assess external validity metrics against random clustering, we created a dataset of 1,000 points randomly divided into 10 groups and then randomly assigned to varying numbers of clusters.

RESOURCE AVAILABILITY

Lead contact

Requests for further information and resources should be directed to and will be fulfilled by the lead contact, Iskra Pollak Dorocic (iskra.pollak@scilifelab.se).

Materials availability

This study did not generate new materials.

Data and code availability

The videos and their associated tracking and clustering, as well as manual labeling datasets used for analysis and benchmarking and video examples of behavioral motifs are publicly available on Zenodo. The accession number for the data reported in this paper is Zenodo: 10.5281/zenodo.14382973.[24]

ACKNOWLEDGMENTS

This study was supported by SciLifeLab and Wenner-Gren Foundation grants to I.P.D., the Polish National Agency for Academic Exchange grant (The Bekker Program - BPN/BEK/2021/1/00152) and the SciLifeLab RED Postdoctoral Fellowship to J.M., and the Wenner-Gren Postdoctoral Fellowship to A.R.C. J.M. would like to thank Tomasz Kojdecki for technical assistance and Marcin Kowynia for discussions regarding the contents of this paper.

AUTHOR CONTRIBUTIONS

J.M. conceptualized the study; implemented DeepLabCut, B-SoiD, and BFA; analyzed and interpreted data; and supervised the work of R.D. and X.L. R.D. implemented and analyzed the VAME data. X.L. implemented and analyzed the Keypoint-MoSeq data. A.R.C. performed the behavioral experiments and collected the data. I.P.D. supervised the study. J.M. wrote the manuscript with input from I.P.D. and A.R.C.

DECLARATION OF INTERESTS

The authors declare no competing interests.

SUPPLEMENTAL INFORMATION

Supplemental information can be found online at https://doi.org/10.1016/j.patter.2025.101237.

Received: December 4, 2024
Revised: February 25, 2025
Accepted: March 31, 2025
Published: April 22, 2025

REFERENCES

1. Jørgensen, S.H., Ejdrup, A.L., Lycas, M.D., Posselt, L.P., Madsen, K.L., Tian, L., Dreyer, J.K., Herborg, F., Sørensen, A.T., and Gether, U. (2023). Behavioral encoding across timescales by region-specific dopamine dynamics. Proc. Natl. Acad. Sci. USA *120*, e2215230120. https://doi.org/10.1073/pnas.2215230120.
2. Flavell, S.W., Gogolla, N., Lovett-Barron, M., and Zelikowsky, M. (2022). The Emergence and Influence of Internal States. Neuron *110*, 2545–2570. https://doi.org/10.1016/J.NEURON.2022.04.030.
3. Pereira, T.D., Shaevitz, J.W., and Murthy, M. (2020). Quantifying behavior to understand the brain. Nat. Neurosci. *23*, 1537–1549. https://doi.org/10.1038/S41593-020-00734-Z.
4. Del Rosario Hernandez, T., Joshi, N.R., Gore, S.V., Kreiling, J.A., and Creton, R. (2024). Combining supervised and unsupervised analyses to quantify behavioral phenotypes and validate therapeutic efficacy in a triple transgenic mouse model of Alzheimer's disease. Biomed. Pharmacother. *181*, 117718. https://doi.org/10.1016/J.BIOPHA.2024.117718.
5. von Ziegler, L.M., Roessler, F.K., Sturman, O., Waag, R., Privitera, M., Duss, S.N., O'Connor, E.C., and Bohacek, J. (2024). Analysis of behavioral flow resolves latent phenotypes. Nat. Methods *21*, 2376–2387. https://doi.org/10.1038/S41592-024-02500-6.
6. Crawley, J.N. (2007). What's Wrong with My Mouse? What's Wrong with My Mouse? (John Wiley & Sons). https://doi.org/10.1002/0470119055.
7. Datta, S.R., Anderson, D.J., Branson, K., Perona, P., and Leifer, A. (2019). Computational Neuroethology: A Call to Action. Neuron *104*, 11–24. https://doi.org/10.1016/J.NEURON.2019.09.038.
8. Mathis, A., Mamidanna, P., Cury, K.M., Abe, T., Murthy, V.N., Mathis, M.W., and Bethge, M. (2018). DeepLabCut: markerless pose estimation of user-defined body parts with deep learning. Nat. Neurosci. *21*, 1281–1289. https://doi.org/10.1038/s41593-018-0209-y.
9. Nath, T., Mathis, A., Chen, A.C., Patel, A., Bethge, M., and Mathis, M.W. (2019). Using DeepLabCut for 3D markerless pose estimation across species and behaviors. Nat. Protoc. *14*, 2152–2176. https://doi.org/10.1038/s41596-019-0176-0.
10. Pereira, T.D., Tabris, N., Matsliah, A., Turner, D.M., Li, J., Ravindranath, S., Papadoyannis, E.S., Normand, E., Deutsch, D.S., Wang, Z.Y., et al. (2022). SLEAP: A deep learning system for multi-animal pose tracking. Nat. Methods *19*, 486–495. https://doi.org/10.1038/s41592-022-01426-1.
11. Anderson, D.J., and Perona, P. (2014). Toward a Science of Computational Ethology. Neuron *84*, 18–31. https://doi.org/10.1016/J.NEURON.2014.09.005.
12. Hsu, A.I., and Yttri, E.A. (2021). B-SOiD, an open-source unsupervised algorithm for identification and fast prediction of behaviors. Nat. Commun. *12*, 5188–5213. https://doi.org/10.1038/s41467-021-25420-x.
13. Luxem, K., Mocellin, P., Fuhrmann, F., Kürsch, J., Miller, S.R., Palop, J.J., Remy, S., and Bauer, P. (2022). Identifying behavioral structure from deep variational embeddings of animal motion. Commun. Biol. *5*, 1267. https://doi.org/10.1038/s42003-022-04080-7.
14. Weinreb, C., Pearl, J.E., Lin, S., Osman, M.A.M., Zhang, L., Annapragada, S., Conlin, E., Hoffmann, R., Makowska, S., Gillis, W.F., et al. (2024). Keypoint-MoSeq: parsing behavior by linking point tracking to pose dynamics. Nat. Methods *21*, 1329–1339. https://doi.org/10.1038/s41592-024-02318-2.
15. Bohic, M., Pattison, L.A., Jhumka, Z.A., Rossi, H., Thackray, J.K., Ricci, M., Mossazghi, N., Foster, W., Ogundare, S., Twomey, C.R., et al. (2023). Mapping the neuroethological signatures of pain, analgesia, and recovery in mice. Neuron *111*, 2811–2830.e8. https://doi.org/10.1016/J.NEURON.2023.06.008.
16. Sturman, O., von Ziegler, L., Schläppi, C., Akyol, F., Privitera, M., Slominski, D., Grimm, C., Thieren, L., Zerbi, V., Grewe, B., and Bohacek, J. (2020). Deep learning-based behavioral analysis reaches human accuracy and is capable of outperforming commercial solutions. Neuropsychopharmacology *45*, 1942–1952. https://doi.org/10.1038/S41386-020-0776-Y.

CellPress
OPEN ACCESS

17. Goodwin, N.L., Choong, J.J., Hwang, S., Pitts, K., Bloom, L., Islam, A., Zhang, Y.Y., Szelenyi, E.R., Tong, X., Newman, E.L., et al. (2024). Simple Behavioral Analysis (SimBA) as a platform for explainable machine learning in behavioral neuroscience. Nat. Neurosci. *27*, 1411–1424. https://doi.org/10.1038/S41593-024-01649-9.

18. Isik, S., and Unal, G. (2023). Open-source software for automated rodent behavioral analysis. Front. Neurosci. *17*, 1149027. https://doi.org/10.3389/FNINS.2023.1149027/BIBTEX.

19. Tang, C., Zhou, Y., Zhao, S., Xie, M., Zhang, R., Long, X., Zhu, L., Lu, Y., Ma, G., and Li, H. (2024). Segmentation tracking and clustering system enables accurate multi-animal tracking of social behaviors. Patterns *5*, 101057. https://doi.org/10.1016/j.patter.2024.101057.

20. Klibaite, U., Li, T., Aldarondo, D., Akoad, J.F., Ölveczky, B.P., and Dunn, T.W. (2025). Mapping the landscape of social behavior. Cell *188*, 2250–2267. https://doi.org/10.1016/j.cell.2025.01.044.

21. Rousseeuw, P.J. (1987). Silhouettes: A graphical aid to the interpretation and validation of cluster analysis. J. Comput. Appl. Math. *20*, 53–65. https://doi.org/10.1016/0377-0427(87)90125-7.

22. Sundqvist, M., Chiquet, J., and Rigaill, G. (2023). Adjusting the adjusted Rand Index: A multinomial story. Comput. Stat. *38*, 327–347. https://doi.org/10.1007/S00180-022-01230-7/METRICS.

23. Romano, S., Vinh, N.X., Bailey, J., and Verspoor, K. (2016). Adjusting for Chance Clustering Comparison Measures. J. Mach. Learn. Res. *17*, 1–32.

24. Mlost, J., Pollak Dorocic, I., Costa, A.R., Dawli, R., and Liu, X. (2025). Data for evaluation of unsupervised learning algorithms for the classification of behavior. Zenodo. https://doi.org/10.5281/zenodo.14382973.

Patterns, Volume *6*

Supplemental information

Evaluation of unsupervised learning algorithms for the classification of behavior from pose estimation data

Jakub Mlost, Rame Dawli, Xuan Liu, Ana Rita Costa, and Iskra Pollak Dorocic

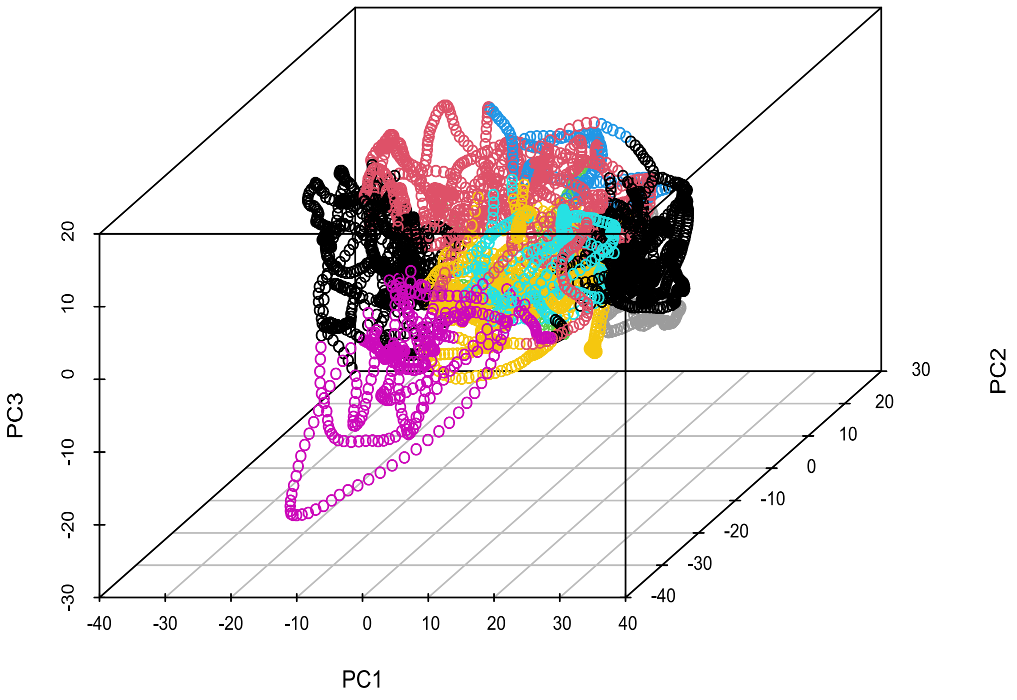

Figure S1. Visualization of the feature space from BFA package after PCA dimensionality reduction and K-means clustering. Related to second paragraph in **Clustering Algorithm** section and Figure 2.

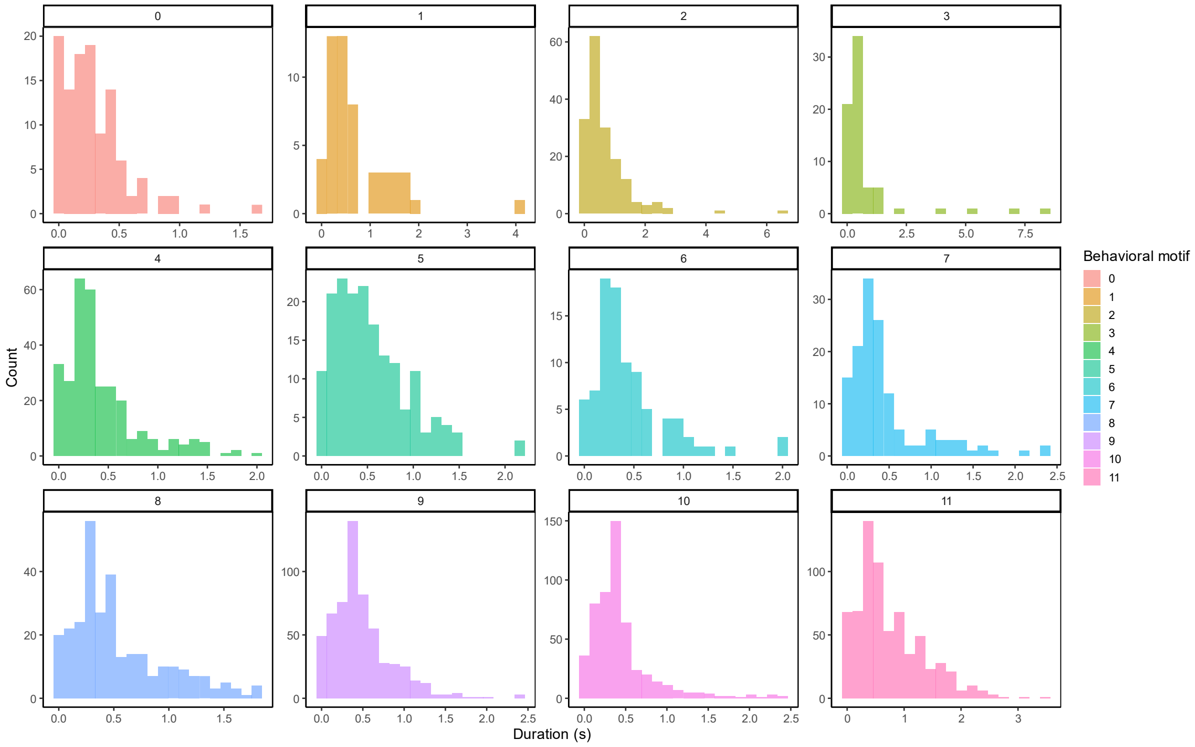

Figure S2. Frequency histograms of number of frames occurring for each behavioral motif produced by B-SOiD. The X-axis presents the duration of the occurrence, while Y axis represents the number of occurrences with a given duration. Values represent distributions across 3 samples of 10-minute recordings. Related to Figure 5.

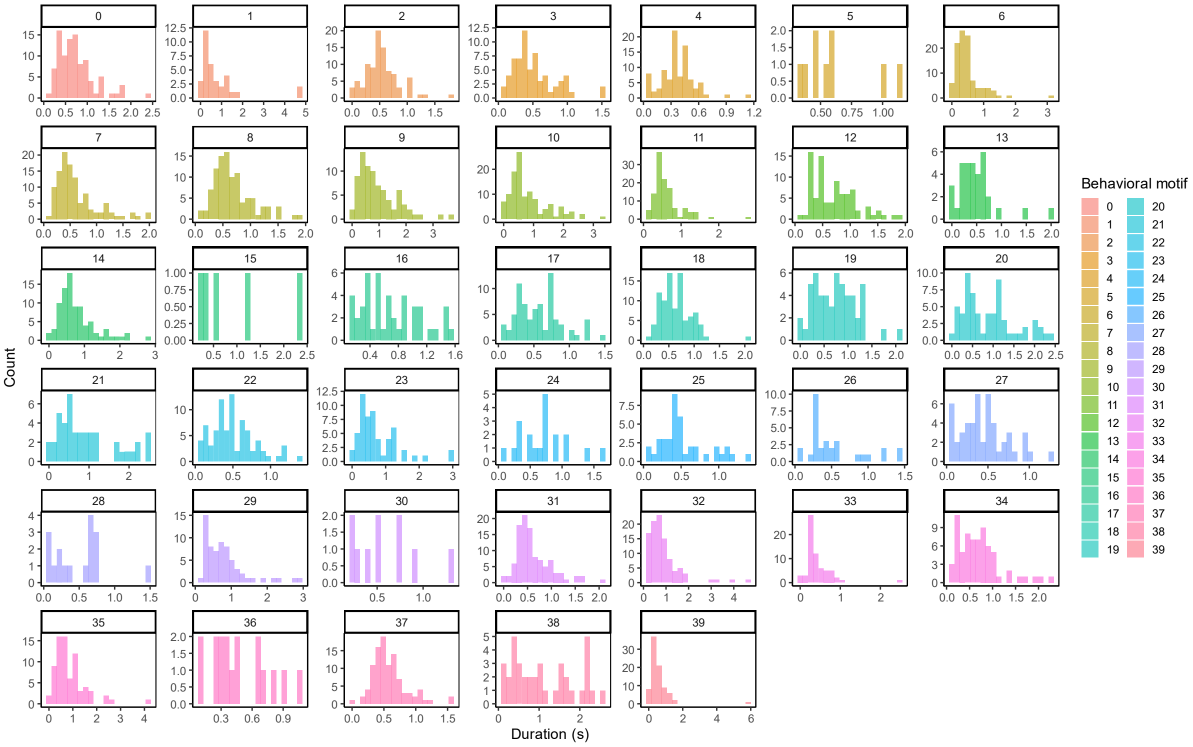

Figure S3. Frequency histograms of number of frames occurring for each behavioral motif produced by VAME. The X-axis presents the duration of the occurrence, while Y axis represents the number of occurrences with a given duration. Values represent distributions across 3 samples of 10-minute recordings. Related to Figure 5.

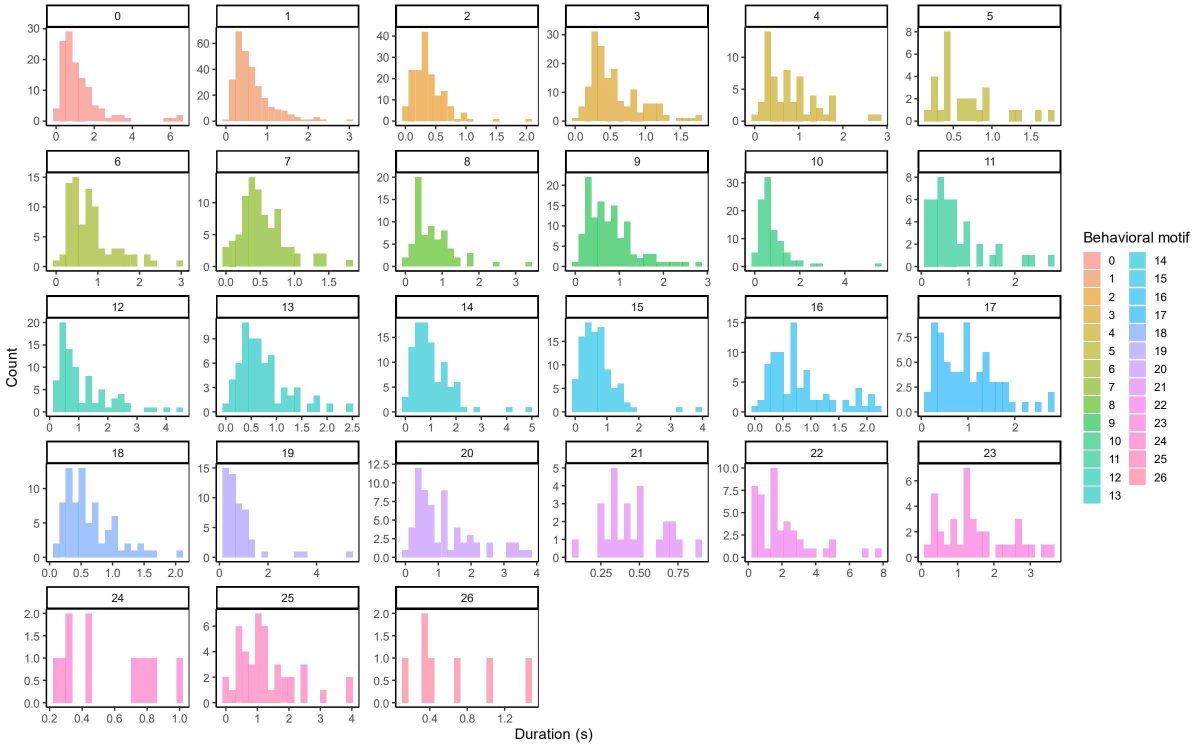

Figure S4. Frequency histograms of number of frames occurring for each behavioral motif produced by Keypoint-MoSeq. The X-axis presents the duration of the occurrence, while Y axis represents the number of occurrences with a given duration. Values represent distributions across 3 samples of 10-minute recordings. Related to Figure 5.

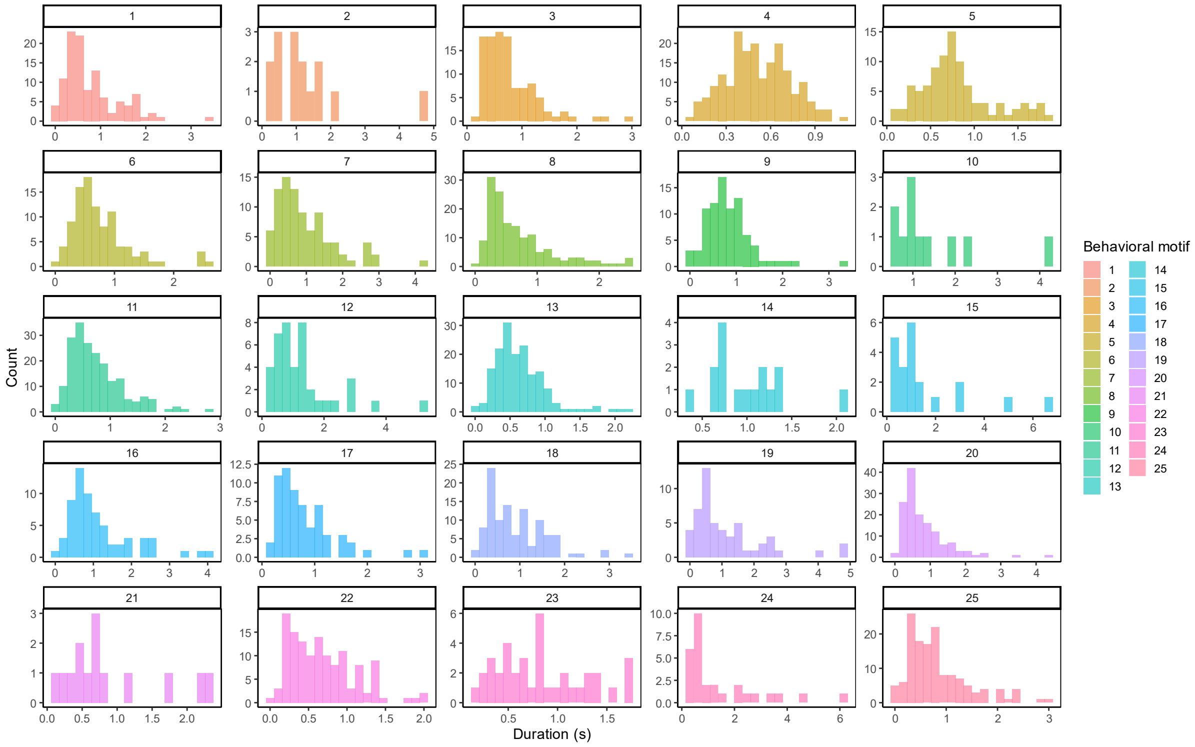

Figure S5. Frequency histograms of number of frames occurring for each behavioral motif produced by BFA. The X-axis presents the duration of the occurrence, while Y axis represents the number of occurrences with a given duration. Values represent distributions across 3 samples of 10-minute recordings. Related to Figure 5.

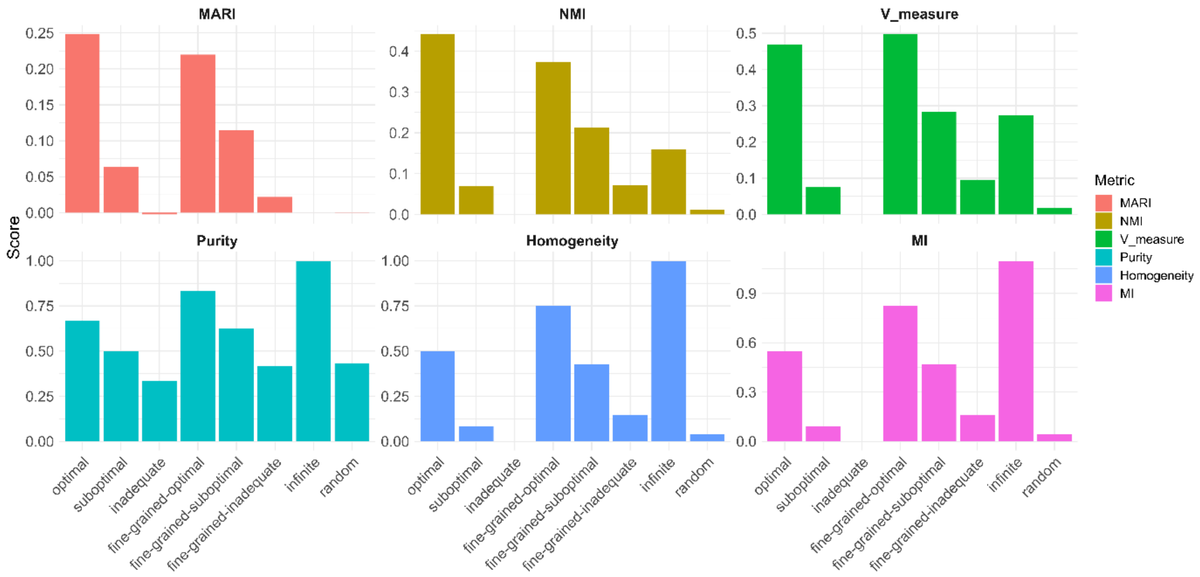

Figure S6. External validity measures scores for MARI (A), NMI (B), V-measure (C), Purity (D), Homogeneity (E) and Mutual Information (F) against mock behavioral data. Optimal solution represents presumed 100% accuracy in consistent clustering of behavioral motifs into more fine grained clusters. Suboptimal, inadequate and invalid solutions represents 75%, 50% and 25% in presumed accuracy against manual labeling data. Fine-grained counterparts are splitting mock manual labeling into a higher number of clusters. Inifite solution has as many clusters as data points. Related to Figure 6.

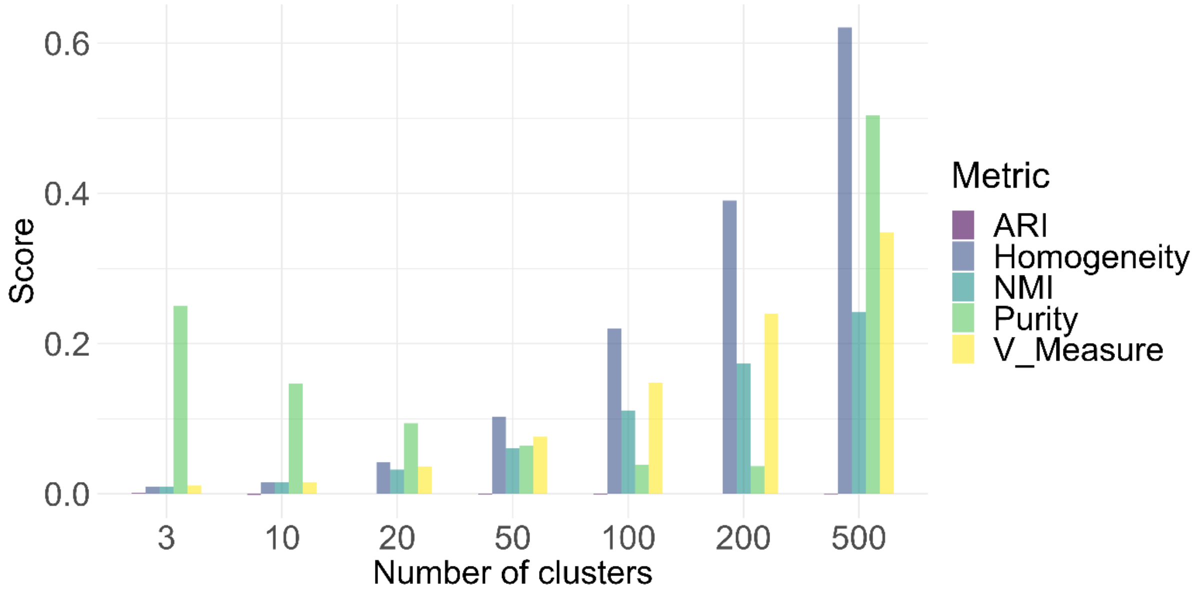

Figure S7. External validity measures scores for a random dataset of 1000 datapoints, split randomly into 10 groups and then assessed against random clustering, related to Figure 6**.**

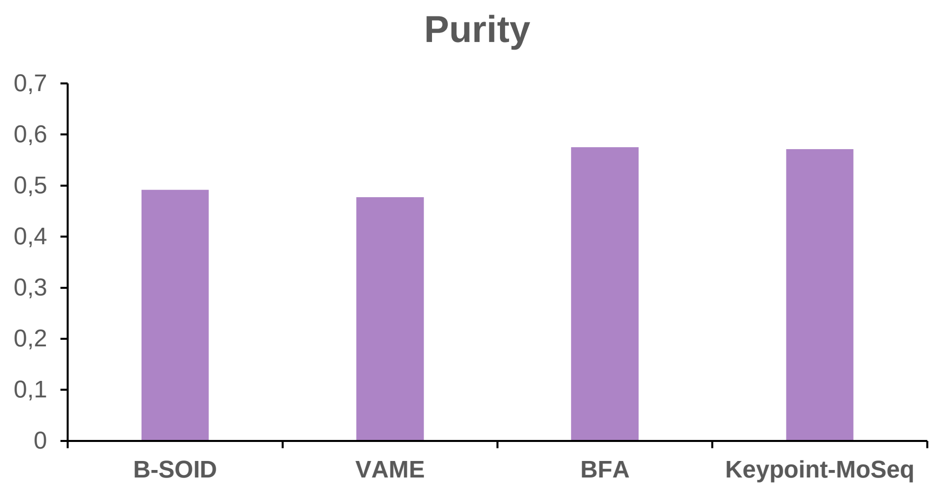

Figure S8. Purity measurement across different clustering algorithms. Positive values denote the best clustering efficacy, related to Figure 6**.**

Score	B-SOiD	VAME	BFA	Keypoint-MoSeq
Silhouette Score	0,240	-0,013	0,017	-0,267
MARI	0,196	0,077	0,157	0,202
Purity	0,491	0,477	0,575	0,571

Table S1. Numerical values for quantitative comparison of different methods, related to Figure 6.

Solution	Purity	MI	MARI	Homogeneity	NMI	V_measure
optimal	0,666	0,549	0,248	0,5	0,442	0,469
suboptimal	0,5	0,093	0,063	0,084	0,068	0,076
inadequate	0,333	0	0	0	0	0
fine-grained-optimal	0,833	0,823	0,22	0,75	0,372	0,498
fine-grained-suboptimal	0,625	0,466	0,115	0,425	0,212	0,283
fine-grained-inadequate	0,417	0,159	0,022	0,145	0,071	0,095
infinite	0,999	1,098	0	0,999	0,159	0,206

Table S2. Numerical values for quantitative comparison of external validation metrics across variable scenarios with mock behavioral data, related to Figure 6.
